# Crystallisation-enhanced bulk hole mobility in phenothiazine-based organic semiconductors

**DOI:** 10.1038/srep46268

**Published:** 2017-04-12

**Authors:** D. B. Shinde, Jagadish K. Salunke, Nuno R. Candeias, Francesca Tinti, Massimo Gazzano, P. P. Wadgaonkar, Arri Priimagi, Nadia Camaioni, Paola Vivo

**Affiliations:** 1 Polymer Science and Engineering Division, CSIR-National Chemical Laboratory, Dr Homi Bhabha Road, Pune 411008, India; 2 Academy of Scientific and Innovative Research, 110025, New Delhi, India; 3 Laboratory of Chemistry and Bioengineering, Tampere University of Technology, P.O. Box 541, FI-33101 Tampere, Finland; 4 Istituto per la Sintesi Organica e la Fotoreattività, Consiglio Nazionale delle Ricerche, via P. Gobetti 101, I-40129 Bologna, Italy

## Abstract

A series of three novel donor-acceptor systems based on C(3)-malononitrile-substituted phenothiazines was synthesised in good overall yields and their thermal, spectroscopic, and electrochemical properties were characterised. The compounds were prepared through a sequence of Ullmann-coupling, Vilsmeier-Haack formylation and Knoevenagel-condensation, followed by Suzuki-coupling reactions for introduction of aryl substitutents at C(7) position of the phenothiazine. The introduction of a donor unit at the C(7) position exhibited a weak impact on the optical and electrochemical characteristics of the compounds and led to amorphous films with bulk hole mobilities in the typical range reported for phenothiazines, despite the higher charge delocalisation as attested by computational studies. In contrast, highly ordered films were formed when using the C(7)-unsubstituted 3-malononitrile phenothiazine, exhibiting an outstanding mobility of 1 × 10^−3^ cm^2^ V^−1^ s^−1^, the highest reported for this class of compounds. Computational conformational analysis of the new phenothizanes suggested that free rotation of the substitutents at the C(7) position suppresses the ordering of the system, thereby hampering suitable packing of the new materials needed for high charge carrier mobility.

The charge carrier mobility plays a significant role when designing new materials for organic electronic applications as it correlates with several key characteristics determining the performance of organic electronic devices. For instance, charge carrier transport directly affects the switching of organic field effect transistors (OFETs), and lower turn-on voltages are observed for organic light emitting diodes (OLEDs) based on materials with high mobility^1^,^2^. The mobility also influences charge carrier dynamics in organic photovoltaics (OPVs)^3^,^4^. A large number of π-conjugated small molecules have been designed and synthesised for OPVs^5^,^6^,^7^,^8^, OLEDs^9^,^10^, and OFETs^11^. With respect to their polymeric counterparts, the benefits of solution-processable small-molecule-based organic semiconductors arise from their high structural flexibility, easy availability, simple preparation and purification, well-defined structure, and batch-to-batch reproducibility^12^,^13^,^14^.

Phenothiazine heterocycle is a well-known electron-rich compound due to the presence of nitrogen and sulphur hetero atoms^15^. Phenothiazines are good electron-donors in photo-excited charge transfer transitions, and display a low and reversible oxidation potential for the generation of a stable radical cation^16^,^17^,^18^,^19^,^20^,^21^. Their ability to suppress molecular aggregation due to butterfly conformation of phenothiazine^22^, together with their attractive hole-transport characteristics have resulted in their use in dye sensitized solar cells (DSSCs) as photosensitizers^22^,^23^,^24^,^25^,^26^,^27^,^28^,^29^,^30^,^31^, as well as in organic thin film transistors and OLEDs^32^,^33^,^34^,^35^. Molecular aggregation and the degree of disorder in phenothiazine films has a strong impact on their optical properties^36^ and better film-forming properties are usually observed in systems for which aggregate formation is less likely to occur^37^,^38^.

The state-of-the-art phenothiazines possess, however, fairly low bulk mobilities^32^,^33^,^34^,^35^, which represents one key reason of the few reports existing on phenothiazines for organic photovoltaics^32^,^39^,^40^,^41^,^42^,^43^. To the best of our knowledge, as the only counter-example, high mobility values for charge-transfer complexes of iodine with N-methylphenothiazine (2:3 donor acceptor ratio) have been reported by Matsunaga in 1960s, where a complex cation radical of phenothiazine was proposed as the conductive species^44^,^45^,^46^,^47^,^48^,^49^,^50^. Finding a phenothiazine derivative combining high mobility, good film-forming properties and high light-harvesting capabilities would therefore be highly desired for various organic electronic applications.

Herein, we report the synthesis and characterisation of three newly designed push-pull *para*-methoxyphenyl *N*-substituted phenothiazine-based organic semiconductors (**O-1**, **O-2** and **O-3**) presented in Fig. 1. Based on previous mobility data for *para*-methoxyphenyl *N*-substituted phenothiazines^29^,^31^, the intramolecular charge transfer (ICT) character of phenothiazines can be improved by introducing substituents at C(3) and C(7) positions^28^. Extending the π-system of the phenothiazine increases ICT and consequently improves its charge transport properties^51^,^52^. Despite the several reports on such approach, the *para*-methoxyphenyl *N*-substituted phenothiazines already studied usually bear a cyanoacrylic moiety at C(3) position^53^,^54^,^55^,^56^,^57^. We envisioned that the substitution of such group with a stronger electron acceptor such as malononitrile would increase the ICT. In order to increase the electron density at the phenothiazine core, alkoxyaryl groups were added at the C(7) position; *para*-methoxyphenyl unit into **O-2** and 6-butoxynaphthalene into **O-3,** to induce solution processability due to bulkier molecular structure. We thoroughly investigated the effect of the donor unit at the C(7) position of 3-malononitrile *para*-methoxyphenyl *N*-substituted phenothiazines on the electrochemical, spectro scopic, and charge-transport properties. To our surprise, molecule **O-1** exhibited not only higher hole-mobility in comparison to **O-2** and **O-3** but, to the best of our knowledge, the highest bulk hole-mobility ever reported in the context of phenothiazine-based small molecules. The relation between the molecular structure, structural (X-ray) analysis, and the outstanding bulk mobility of **O-1** with respect to the other two proposed phenothiazines (**O-2**, **O-3**) is also discussed in detail.

## Results and Discussion

### Synthesis

**Figure 2:**
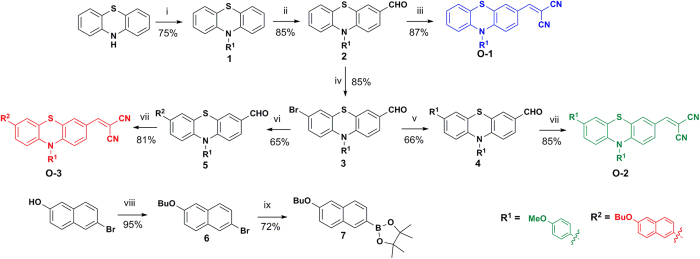
Synthesis of push-pull organic semiconductors **O-1**, **O-2** and **O-3**. Reagents: (i) 4-Iodoanisole, Cu, K_2_CO_3_, TEGDME, 180 °C; (ii) POCl_3_, DMF, C_2_H_4_Cl_2_, 80 °C; (iii and (vii) CH_2_(CN)_2_, Piperidine, CHCl_3_, reflux; (iv) N-Bromosuccinimide, CHCl_3_, rt; (v) 4-methoxyphenyl boronic acid, Pd(PPh_3_)_4_, K_2_CO_3_ (2M), THF; (vi) 2-(6-butoxynaphthalen-2-yl)-4,4,5,5-tetramethyl-1,3,2-dioxoborolane, Pd(PPh_3_)_4_, K_2_CO_3_ (2M), THF; (viii) 1-bromobutane, KOH, DMSO, rt; (ix) bis(pinacolato)diboron, Pd(dppf)Cl_2_, KOAc, 1,4-dioxane, 80 °C.

The synthesis route of **O-1**, **O-2**, and **O-3** is depicted in Fig. 2. 10-(4-Methoxyphenyl)-10*H*-phenothiazine 1 was prepared (75% yield) in one-step reaction via Ullmann-coupling of 4-iodoanisole and commercially available phenothiazine, according to a previously reported procedure^9^. The subsequent Vilsmeier-Haack formylation of 1 in 1,2-dichloroethane produced its mono-formyl derivative 2 in 85% yield. The Knoevenagel-condensation reaction of 2 with an excess amount of malononitrile in the presence of piperidine and chloroform afforded **O-1** in 87% yield. Bromination of 2 using *N*-bromosuccinimide (NBS) in chloroform produced its bromo-derivative 3 in 85% yield. Furthermore, Suzuki-coupling reaction of 3 with two different electron donors, 4-methoxyphenyl boronic acid and previously synthesized 2-(6-butoxynaphthalen-2-yl)-4,4,5,5-tetramethyl-1,3,2-dioxaborolane 7 in presence of catalyst Pd(PPh_3_)_4_ in inert atmosphere afforded compounds 4 and 5 in 66 and 65% of yields, respectively.

Finally, introduction of malononitrile to obtain **O-2** and **O-3** was achieved as described above for **O-1** in 85 and 81% yields, respectively. All the compounds were easily soluble in common organic solvents, which allowed their purification by column chromatography. All the reaction intermediates, as well as the final products, were characterized and confirmed by ^1^H NMR, ^13^C NMR and high-resolution mass spectroscopy (see Supplementary Information, SI, Figs S1–S11).

### UV-vis spectroscopy

**Figure 3:**
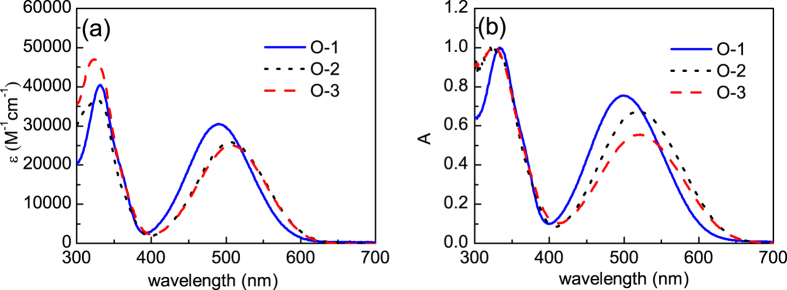
(**a**) molar absorptivity (ε) of **O-1**, **O-2** and **O-3** in chloroform solution; (**b**) absorption spectra of freshly prepared films of the target compounds.

The UV-vis absorption spectra (in units of molar absorptivity, ε) of **O-1**, **O-2**, and **O-3** in chloroform solution (concentration 10^−5^ M), together with the absorption spectra of thin films normalised at the Soret-transition (S0-S2) absorption maximum, are shown in Fig. 3 and summarised in Table 1. The absorption spectra of the three molecules show two distinct bands: one at 320–330 nm and another at 490–510 nm. The former corresponds to π-π* electronic transition of the chromophores (Soret band). The latter is attributed to the intra- molecular charge transfer (ICT) from the donor to the acceptor moieties (Q band). As can be seen from Fig. 3, the Soret and Q bands are comparable, with slightly higher intensity for the Soret bands. Furthermore, for compounds **O-2** and **O-3**, the absorption maxima are overlapping (shift in λ_max_ of 2 nm), while **O-1** absorption peaks are slightly blue-shifted by 12–13 nm. The red-shift of the absorption maxima of **O-2** and **O-3** with respect to **O-1** can be attributed to their slightly more extended conjugation. The absorption spectra of the films spin-coated onto glass substrates are bathocromically-shifted by 10–12 nm as compared to the spectra measured in solution, most likely due to chromophore-chromophore intermolecular interactions^58^.

### Electrochemical analysis

The electrochemical properties of **O-1**, **O-2**, and **O-3** were determined by differential pulse voltammetry (DPV) in dichloromethane solution. The voltammograms, *i.e.* changes in current-voltage curves, are presented in SI with respect to the ferrocene couple (Fig. S13). The highest occupied molecular orbital (HOMO) levels, corresponding to the ionization potentials of the materials, were derived from DPV and are presented in Table 1. All the compounds exhibited similar electrochemical properties: the HOMO values lie in the range of −5.24 to −5.29 eV. Since no clear reduction peaks were appreciated from the voltammograms of Fig. S12, the LUMO values were derived from the optical bandgap (E_g_^opt^) reported in Table 1, as explained in the Methods section.

### Thermal analysis

The thermal properties of **O-1**, **O-2**, and **O-3** were investigated with differential scanning calorimetry (DSC) and thermogravimetric analysis (TGA). Interestingly, given its lowest molecular weight in the series, DSC data in Fig. S13a (SI) showed a higher melting point for **O-1** (199 °C) compared to **O-2** (194 °C) and **O-3** (163 °C). This can be attributed to its molecular packing and tendency towards crystallisation, as detailed later on. In case of **O-3**, large drop in the melting point was observed as compared to **O-2**, probably due to the presence of the butyl chain. The thermal decomposition temperatures (T_d_) of the compounds were obtained from the TGA curves shown in Fig. S13b (SI), with an increasing trend with increasing molecular weight, *i.e.* **O-1 **< **O-2 **< **O-3**. The results of the thermal characterisation, summarized in Table 1, support the suitability of these materials for organic electronic device fabrication^59^.

### Hole mobility characterisation

The bulk hole mobility in films of **O-1**, **O-2**, and **O-3** was investigated by means of impedance spectroscopy^60^ applied to hole-only devices. Upon injecting charge carriers, by applying a dc bias superimposed to small harmonic voltage modulation, dramatic changes were observed in the impedance spectra^61^,^62^. The transit time of charge carriers τ can be inferred from the peak frequency of the negative differential subsceptance^61^,^62^ or from the peak frequency of the imaginary part of impedance (ImZ)^63^. For dispersive charge transport, the latter method is considered more convenient. Indeed, even in high dispersion conditions, clear peaks are observed in the ImZ spectrum^63^.

**Figure 4:**
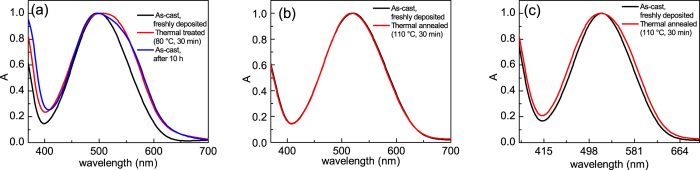
Normalised absorption spectra of **O-1** (**a**), **O-2** (**b**) and **O-3** (**c**) films, spin-coated from chlorobenzene solutions. Spectra of as-cast freshly deposited films, of as-cast films after 10 h at room temperature, and of thermally annealed films are shown.

The phenothiazine films for impedance spectroscopy measurements were deposited by spin-coating from chlorobenzene solutions. Differently from **O-2** and **O-3**, the optical properties of **O-1** films underwent a clear and spontaneous variation even at room temperature, leading in some hours to a change of colour visible to the naked eye. Moreover, the spectral variation of **O-1** films was greatly accelerated by the thermal treatment applied to remove the excess solvent. In Fig. 4a the absorption spectrum of a freshly deposited **O-1** film is compared with that obtained after thermal annealing at 80 °C for 30 min (in an Ar-filled glove-box) and with that of a film stored at ambient temperature for around 10 h. In the latter two cases, the spectra are similar and broader compared to the spectrum of the freshly deposited **O-1** film, with an enhanced absorption toward lower energies. The spontaneous change in the spectral properties of **O-1** films at room temperature and upon thermal annealing indicates enhanced intermolecular electronic interactions, possibly due to evolution towards a more ordered arrangement of **O-1** molecules in the solid state. The described behaviour was not observed for **O-2** and **O-3** films, which preserved to a large extent the original spectral features upon thermal treatment at 110 °C for 30 min (Fig. 4b and c). It should be noted that a lower temperature (80 °C), was applied for the thermal annealing of **O-1** films. That choice was motivated by the deterioration of **O-1** film compactness (appearance of pin-holes and cracks) upon heating above 80 °C, making them useless in the preparation of sandwich-type devices.

**Figure 5:**
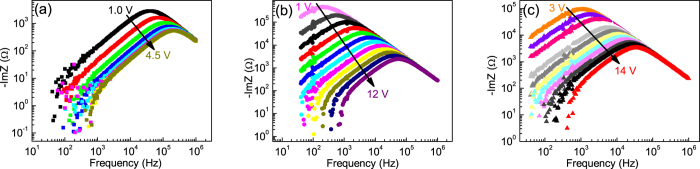
ImZ as a function of frequency for hole-only devices made of **O-1** (**a**), **O-2** (**b**) and **O-3** (**c**) films at different values of the dc voltage (the arrows indicate increasing voltage). Film thickness: 920 nm, 575 nm and 470 nm for **O-1**, **O-2** and **O-3**, respectively.

Hole-only devices for charge carrier mobility investigation were prepared with thermally annealed films, at 80 °C for **O-1**, and at 110 °C for **O-2** and **O-3**. The frequency dependence of ImZ for typical hole-only devices is shown in Fig. 5 for different values of the dc voltage. Clear peaks were observed for all molecules, shifting towards higher frequencies as the voltage increased. The transit time of charge carriers was obtained from the peak frequency through *τ* = *k*·*τ*_*p*_, where *τ*_*p*_ is the time constant corresponding to the peak frequency and *k* a constant dependent on the dispersion degree^63^. The value of 0.44 was assumed for *k*, reported for a moderate degree of dispersion^63^.

**Figure 6:**
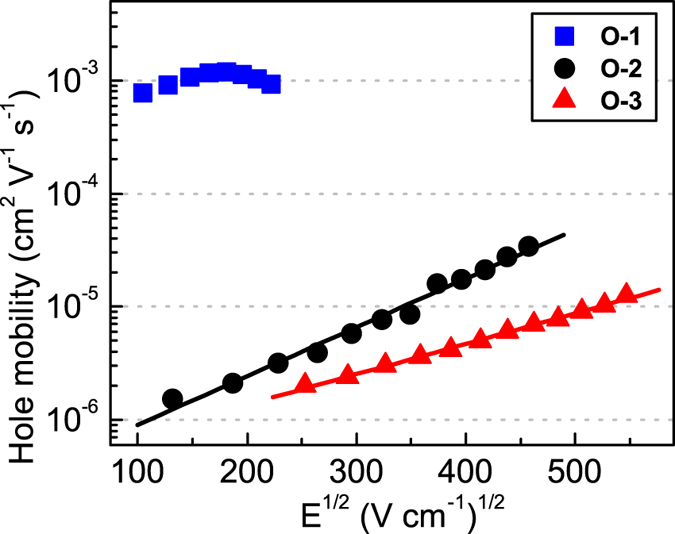
Bulk hole mobility of **O-1**, **O-2** and **O-3** as a function of the square root of electric field. For **O-2** and **O-3** the lines indicate the linear fit to the experimental data.

The hole mobility *μ* was calculated by using the well-known expression 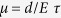, where *d* is the film thickness and *E* the electric field. The obtained values are displayed in Fig. 6 as a function of *E*^1/2^. An outstanding bulk mobility of positive charge carriers was achieved for **O-1** films, with values around 1 × 10^−3^ cm^2^ V^−1^ s^−1^. For **O-2** and **O-3**, *μ* dropped dramatically, by three orders of magnitude, as compared to **O-1** films, with values on the order of 10^−6^ cm^2^ V^−1^ s^−1^ in the same field range. The linear trend of mobility data for **O-2** and **O-3** shown in Fig. 6 indicates a Poole-Frenkel-type behaviour of mobility^64^,^65^:


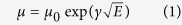


where *μ*_*0*_ denotes the mobility at zero field and *γ* is the parameter describing how strong is the field dependence. The parameters for the Poole-Frenkel fit to the mobility data of **O-2** film are *μ*_*0*_ = 3.4 × 10^−7^ cm^2^ V^−1^ s^−1^ and *γ* = 9.9 ×^ ^10^−3^ (V cm^−1^)^−1/2^, while *μ*_*0*_ = 4.0 ×^ ^10^−7^ cm^2^ V^−1^ s^−1^ and *γ* = 6.2 ×^ ^10^−3^ (V cm^−1^)^−1/2^ were obtained for **O-3**.

**Figure 7:**
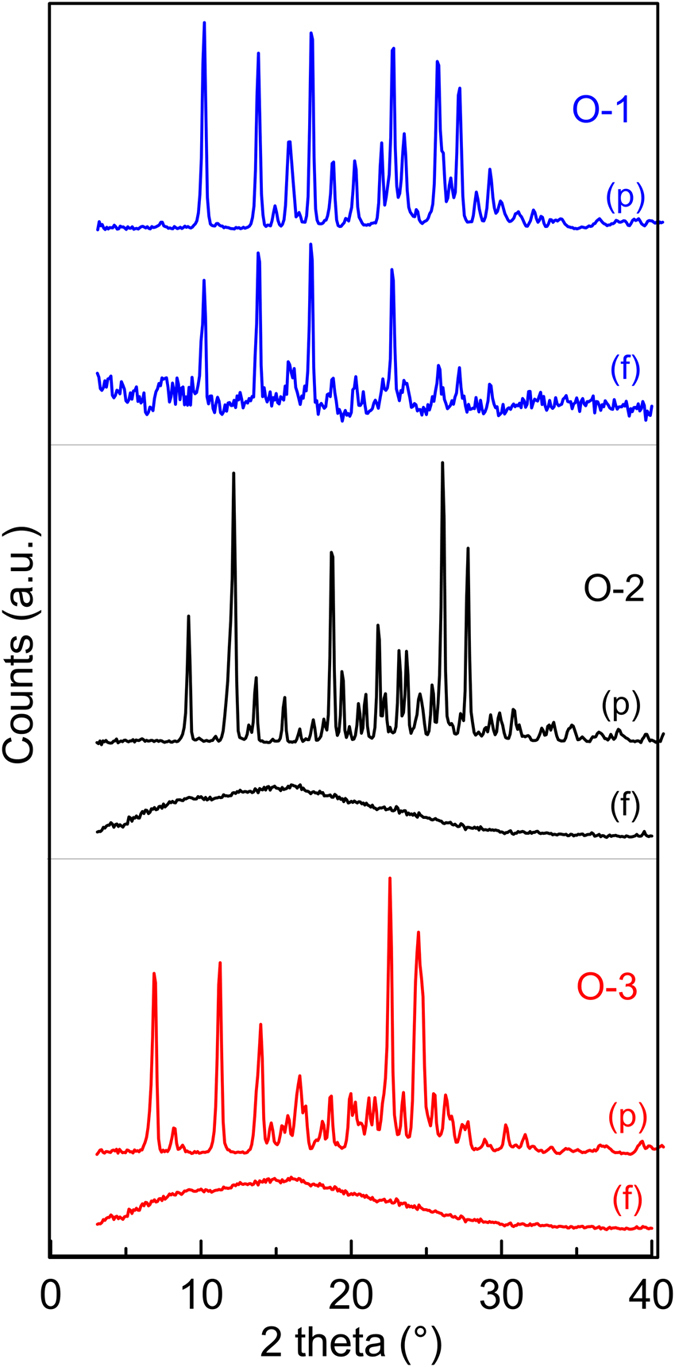
XRD patterns of powder samples (p) and film samples (f). All patterns are subtracted for the background of the substrates in order to enhance the presence of halos due to the amorphous component. The recorded patterns are reported in Figure S14. Film samples were prepared and thermally treated in the same conditions used for the preparation of hole-only devices.

To clarify the significant difference in charge transport properties of **O-1** with respect to **O-2** and **O-3**, we performed powder x-ray diffraction (XRD) analysis. The XRD patterns recorded on (i) film samples, prepared and treated in the same conditions used for the preparation of hole-only devices, and (ii) powder samples are compared in Fig. 7. The sharp peaks of the XRD patterns of the powder samples indicate a high degree of crystallinity for each of the three phenothiazines. For film samples, on the other hand, the XRD pattern of **O-1** indicates the presence of crystalline material also in the film, in the same phase as in the powder, while amorphous patterns were obtained for the **O-2** and **O-3** films. The presence of crystalline phase is well in line with the annealing-induced spectral changes observed in **O-1** films (Fig. 4a) and the absence of such changes for **O-2** and **O-3**.

XRD investigations of the films confirm that the outstanding bulk hole mobility in **O-1** films can be ascribed to the presence of crystalline phases, completely absent in the layers deposited from the other two phenothiazines. The high degree of ordering of **O-1** is probably due to its more compact and less twisted molecular structure as compared to **O-2** and **O-3.** The latter ones have bulkier substituents in the C(7) position, which are able to freely rotate (see the computational analysis), thereby decreasing the system order. Despite some exceptions on increased charge mobility with decreased π-π stacking^66^, shorter π-π stacking distances typically leads to higher charge carrier mobility^1^,^67^,^68^. Hence, a better π-π stacking in case of **O-1** could be responsible for its high hole mobility (*ca*. 10^−3^ cm^2^ V^−1^ s^−1^) whereas the more twisted nature of **O-2** and **O-3** leads to poor mobilities (*ca*. 10^−6^ cm^2^ V^−1^ s^−1^). These low mobility values are comparable to those reported in the literature for other solution-deposited^32^,^33^,^34^,^35^,^69^ and thermally evaporated^70^ phenothiazine derivatives. To the best of our knowledge, the mobility of **O-1** is the highest reported for phenothiazine-based small molecules, without complexation with iodine.

### Computational analysis

In order to get further insight into the electronic nature of the newly synthesised phenothiazines and to support our suggestion for the higher hole mobility of **O-1**, the compounds were studied by Density Functional Theory methods. Compounds **O-1** and **O-2** were optimised at the PBE1PBE/6-31G** level of theory, as well as **O-3**, simplified by replacement of the *n*-butyl chain with a methyl (**O-3(Me)**), and a detailed computational analysis can be found in the SI. The conformational analysis of the three phenothiazines studied showed that in the ground state the preferential conformations of the compounds are slightly bent in the phenothiazine heterocyclic ring and adopt a butterfly shape. The presence of different substituents at the C(7) position of the phenothiazine has little or no effect on the dihedral angles made by the two aromatic rings, being about 19–20° for the three molecules studied. The introduction of aryl substituents at C(7) position of phenothiazines **O-2** and **O-3(Me)** does not encumber the resonance between the substituents and the phenothiazine ring, although such substituents are preferentially placed ca. 34° out of the plane. The rotation of these substituents about the C-C bonds requires only 3.4 kcal/mol, which might explain the above-mentioned disorder of these systems and the absence of π-π stacking suggested for **O-1**.

**Figure 8:**
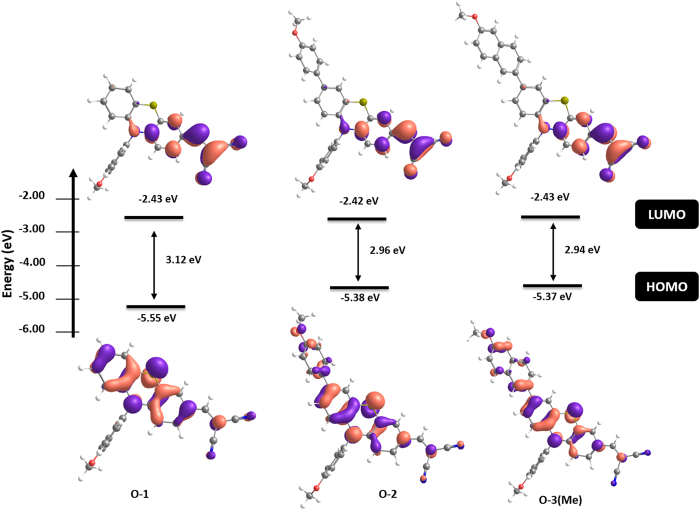
Energy levels and electron distribution for frontier molecular orbitals of **O-1**, **O-2** and **O-3**(Me) calculated with DFT at PBE1PBE/6-31G** level of theory (isosurface value = 0.04).

The electronic distribution in HOMO and LUMO levels are presented in Fig. 8. The electron density distribution in the LUMO of the phenothiazines is mainly localised on the malononitrile end group and the adjacent benzene ring, while in **O-2** and **O-3(Me)** the HOMO orbitals are delocalised over the phenothiazine moiety and its C(7) substituents. Clearly, introduction of electron-rich substituents in the C(7) position of the phenothiazine increases their HOMO energy while keeping the energy of LUMO orbitals localised on the acceptor end. The λ_max_ found reflects the observed experimental trends, although the absolute values for the excitation energies are overestimated by the method (by *ca.* 0.9–1.1 eV). As similar electronic features were determined for these three phenothiazines, the higher charge mobility of **O-1** should not be attributed to the modification of the electron density by introduction of substituents at C(7), but to the lower degrees of freedom of **O-1**.

### Conclusions

A series of new donor-acceptor systems based on C(7)-substituted phenothiazine derivatives was synthesised with good overall yields. The presence of crystalline phases in spin-coated films prepared with the unsubstituted phenothiazine **O-1**, arising from the chemical structure of the compound, likely leads to excellent π-π stacking. While the introduction of bulky *para*-methoxyphenyl and 6-butoxynaphthyl terminal groups in C(7) position of **O-2** and **O-3** has a weak impact on the optical and electrochemical characteristics of the compounds, it has a strong influence on the structural properties of the related spin-coated thin films. In fact, disorder is introduced into **O-2** and **O-3** compounds, by making them more twisted, as the rotation of the bulky substituents requires as little as 3.4 kcal/mol. This explains the observed amorphous nature of **O-2** and **O-3** films. The values of the bulk hole mobility of **O-2** and **O-3** samples (in order of 10^−6^ cm^2^ V^−1^ s^−1^) lie in the typical range of state-of-the-art phenothiazines, while **O-1** shows an outstanding mobility in the order of 10^−3^ cm^2^ V^−1^ s^−1^, the highest reported for phenothiazine-based materials. The achievements of this study provide a strong motivation to further design phenothiazine-based donor-acceptor systems with high bulk mobility, to be adopted as hole-transport materials for organic electronics. Work in this direction is underway and will be reported in future publications.

## Methods

### Synthesis

All chemicals were purchased from Sigma Aldrich and were used without further purification. The chemical reactions were carried out using round-bottom flasks and Schlenk tubes under argon or nitrogen environment. ^1^H NMR and ^13^C NMR spectra were recorded on Bruker spectrometer (^1^H, 400 MHz and ^13^C, 100 MHz) in CDCl_3_. Chemical shifts were given in ppm with reference to tetramethyl silane (TMS). Molecular mass was determined by using Thermo Scientific Q-Exactive Accela 1250 pump mass spectrometer. Purification of the products was carried out by column chromatography on silica gel 60 (Sigma Aldrich) with mesh size 0.040–0.063 mm. The complete details on the synthesis of **O-1**, **O-2**, **O-3** and their intermediate products are provided in SI.

### Spectroscopic measurements

The steady-state absorption spectra were measured with a Shimadzu UV-3600 UV/Vis/NIR spectrophotometer both in solution and in thin films. Thin films of the three compounds were deposited by spin-coating (WS-400B-6NPP/LITE, Laurell Technologies) from CHCl_3_ solution (1700 rpm, 1 min) onto clean glass substrates.

### Electrochemical Measurements

Differential pulse voltammetry (DPV), for HOMO/LUMO determination of the target compounds, was performed by employing a potentiostat (Compact-Stat, Ivium Technologies) and a three-electrode cell configuration. Dry tetrabutyl ammonium tetrafluoroborate in dichloromethane (0.1 M) was the supporting electrolyte, glass platinum electrode the working electrode, Pt wire the counter-electrode, and Ag/AgCl wire the pseudo-reference electrode. Ferrocene/ferrocenium (Fc/Fc+) couple was used as an internal standard reference to scale the measured potentials against the vacuum level^17^. All solutions were deoxygenated with N_2_ prior to each experiment. The measurements were carried out between −2.2 V and 2.0 V, scanning in both directions with scan rate of 50 mV/s The HOMO energy levels were calculated from the oxidation potentials observed from the DPV curves according to the equation:


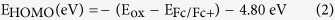


where E_ox_ is the oxidation potential of the sample and E_Fc/Fc+_ the potential of ferrocene. E_ox_ and E_Fc/Fc+_ are both referred against the Ag/AgCl reference electrode. The value −4.80 eV is the energy level of ferrocene against vacuum^71^. The LUMO levels were derived from the optical bandgap, since no clear reduction peaks could be appreciated from the DPV curves:


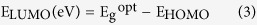


### Charge carrier mobility measurements

Hole-only devices were prepared in the sandwiched structure ITO/PEDOT:PSS/phenothiazine/MoO_3_/Au, where ITO is indium tin oxide and PEDOT:PSS is poly(3,4-ethylenedioxythiophene)/polystyrene sulphonic acid (CLEVIOS P VP AI 4083, H.C. Starck). ITO-coated glass substrates were first cleaned in detergent and water, then ultrasonicated in acetone and isopropyl alcohol for 15 min each. The layer of PEDOT:PSS (~40 nm) was spin-coated at 4000 rpm onto the ITO-glass substrates, and baked in an oven at 120 °C for 10 min. The phenothiazine layers were deposited under ambient conditions by spin-coating (300 rpm) from chlorobenzene solutions (65–70 g/l). After the deposition, the films were transferred in an Ar-filled glove-box, where a thermal treatment was applied to remove the excess solvent before the top contact deposition. The MoO_3_ layer (5 nm) and the Au top electrode (100 nm) were thermally evaporated at a base pressure of 4 × 10^−6^ mbar through a shadow mask defining a device active area of 8 mm^2^.

The electrical characterization of the devices was carried out at room temperature in glove-box. Impedance spectroscopy measurements were conducted using an Agilent 4294A impedance analyzer. The impedance measurements were done in the frequency range 40 Hz–1.4 MHz, with an amplitude of the harmonic voltage modulation of 20 mV. The dc bias was varied in the range 0 V–14 V.

### XRD investigation

The powder samples were analysed in an aluminium sample-holder 0.2 mm deep. The film samples were deposited on quartz plates by using the same conditions used for the preparation of the phenothiazine layers for hole-only devices. After the deposition, the film samples were thermally annealed in the same conditions used for the related devices. The XRD scans were performed in the interval 5–60° (2theta) with a PANalytical X’Pert diffractometer in reflection geometry equipped with a copper anode (λmean = 1.5418 Å) and a fast X’Celerator detector.

### Computational Calculations

Full geometrical optimizations were performed by Density Functional Theory^72^ using PBE1PBE/6-31G** hybrid functional and influence of solvent (CHCl_3_) was taken into account by the application of the polarized continuum model using the Gaussian09 program. The vertical excitations of the optimized ground state geometries were further calculated with the time-dependent DFT (TDDFT)^73^ method and the linear response PCM (CHCl_3_ as solvent) to determine the first-six transitions. A full account on the computational details and the corresponding reference list are presented as SI.

## Additional Information

**How to cite this article**: Shinde, D. B. *et al*. Crystallisation-enhanced bulk hole mobility in phenothiazine-based organic semiconductors. *Sci. Rep.* **7**, 46268; doi: 10.1038/srep46268 (2017).

**Publisher's note:** Springer Nature remains neutral with regard to jurisdictional claims in published maps and institutional affiliations.

## Supplementary Material

Supplementary Information

## Figures and Tables

**Figure 1:**
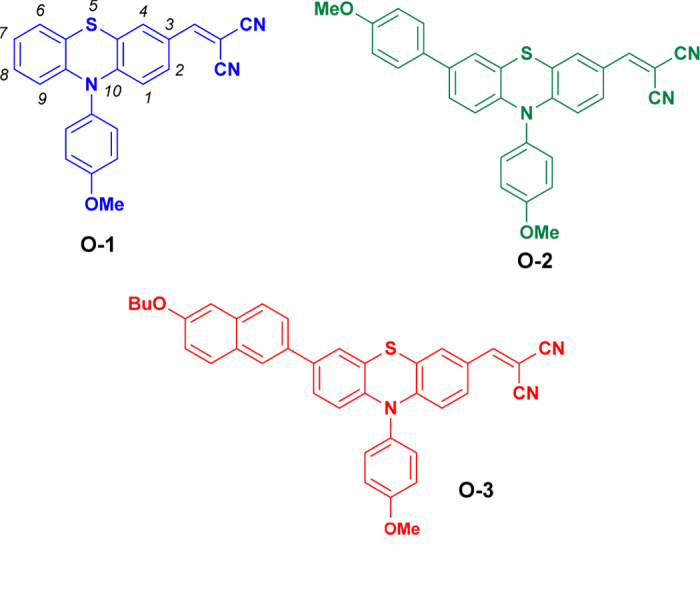
Chemical structures of the phenothiazine derivatives **O-1**, **O-2**, and **O-3**.

**Table 1:** Optical, electrochemical, and thermal characterisation of **O-1**, **O-2**, and **O-3**.

Compound	λ_max,_ (nm)^a^	ε at λ_max_ (M^−1^cm^−1^)	λ_max_ (nm)^b^	E_g_^opt^ (eV)	E_g_ (eV)	HOMO^DPV^ (eV)^c^	LUMO^opt^ (eV)^d^	T_d_/T_m_ (°C)^e^
**O-1**	330, 490	30 437	333, 500	2.06	2.09	−5.29	−3.16	302/199
**O-2**	331, 507	25 757	324, 518	2.02	2.08	−5.24	−3.23	386/194
**O-3**	523, 509	24 957	325, 521	2.00	2.08	−5.24	−3.27	404/163

Wavelengths corresponding to the absorption maxima in ^a^10^−5^ M chloroform solution and in ^b^film samples, spin-coated from chloroform solution. ^c^HOMO^DPV^ values were determined experimentally by DPV measurements. ^d^LUMO^opt^ is derived from optical bandgap (Eg), *i.e.* from the onset of the absorption spectrum (E = hc/λ). LUMO^opt^ = E_g_^opt^ − HOMO^DPV^. ^e^T_d_ is determined from TGA (see SI), T_m_ measured with open capillary method.

## References

[b1] Coropceanu V. . Charge transport in organic semiconductors. Chem. Rev. 107, 926–952 (2007). 17378615 10.1021/cr050140x

[b2] Sirringhaus H., Tessler N. & Friend R. H. Integrated optoelectronic devices based on conjugated polymers. Science 280, 1741–1744 (1998). 9624049 10.1126/science.280.5370.1741

[b3] Mandoc M. M., Koster L. J. A. & Blom P. W. M. Optimum charge carrier mobility in organic solar cells. Appl. Phys. Lett. 90. 133504 (2007).

[b4] Yoshikawa S., Saeki A., Saito M., Osaka I. & Seki S. On the role of local charge carrier mobility in the charge separation mechanism of organic photovoltaics. Phys. Chem. Chem. Phys. 17, 17778–17784 (2015). 26084482 10.1039/c5cp01604e

[b5] Mishra M. & Bäuerle P. Small molecule organic semiconductors on the move: promises for future solar energy technology. Angew. Chemie Int. Ed. 51, 2020–2067 (2012). 10.1002/anie.201102326 22344682

[b6] Liu Y. . Solution-processed small-molecule solar cells: breaking the 10% power conversion efficiency. Sci. Rep. 3, 3356 (2013). 24285006 10.1038/srep03356 PMC3842540

[b7] Roncali J., Leriche P. & Blanchard P. Molecular Materials for Organic Photovoltaics: Small is Beautiful. Adv. Mater. 26, 3821 (2014). 24687246 10.1002/adma.201305999

[b8] Mohamed S., Demeter D., Laffitte J.-A., Blanchard P. & Roncali J. Structure-properties relationships in triarylamine-based donor-acceptor molecules containing naphtyl groups as donor material for organic solar cells. Sci. Rep. 5, 9031 (2015). 25761773 10.1038/srep09031 PMC4356976

[b9] Salunke J. K. . Phenothiazine and carbazole substituted pyrene based electroluminescent organic semiconductors for OLED devices. J. Mater. Chem. C 4, 1009–1018 (2016).

[b10] Yu T. C., Liu L. L., Xie Z. Q. & Ma Y. G. Progress in small-molecule luminescent materials for organic light-emitting diodes. Sci. China-Chemistry 58, 907–915 (2015).

[b11] Wu W., Liu Y. & Zhu D. π-Conjugated molecules with fused rings for organic field-effect transistors: design, synthesis and applications. Chem. Soc. Rev. 39, 1489–1502 (2010). 20419204 10.1039/b813123f

[b12] Yan H. . A high-mobility electron-transporting polymer for printed transistors. Nature 457, 679–686 (2009). 19158674 10.1038/nature07727

[b13] Yu G., Gao J., Hummelen J. C., Wudl F. & Heeger A. J. Polymer Photovoltaic Cells: Enhanced Efficiencies via a Network of Internal Donor-Acceptor Heterojunctions. Science 270, 1789–1791 (1995).

[b14] Bazan G. & Bryce M. R. Themed issue on small molecules and monodisperse oligomers for organic electronics. J. Mater. Chem. C 4, 3675–3676 (2016).

[b15] Wu T. Y. . Synthesis, Characterization and Photovoltaic Properties of Di-Anchoring Organic Dyes. J. Braz. Chem. Soc. 22, 780–789 (2011).

[b16] Krämer C. S., Zeitler K. & Müller T. J. J. First Synthesis and Electronic Properties of (Hetero)aryl Bridged and Directly Linked Redox Active Phenothiazinyl Dyads and Triads. Tetrahedron Lett. 42, 8619–8624 (2001).

[b17] Sailer M., Franz A. W. & Müller T. J. J. Synthesis and Electronic Properties of Monodisperse Oligophenothiazines. Chem. - A Eur. J. 14, 2602–2614 (2008). 10.1002/chem.200701341 18213672

[b18] Memminger K., Oeser T. & Müller T. J. J. Phenothiazinophanes: Synthesis, Structure, and Intramolecular Electronic Communication. Org. Lett. 10, 2797–2800 (2008). 18533671 10.1021/ol800920d

[b19] Oka H. Synthesis and through-bond spin interaction of stable 1,3-phenylene linked poly(phenothiazine cation radical). J. Mater. Chem. 18, 1927–1934 (2008).

[b20] Hong B. C., Dange N. S., Ding C. F. & Liao J. H. Organocatalytic Michael–Knoevenagel–Hetero-Diels–Alder Reactions: An Efficient Asymmetric One-Pot Strategy to Isochromene Pyrimidinedione Derivatives. Org. Lett. 14, 448–451 (2012). 22195677 10.1021/ol202877m

[b21] Okamoto T. . Remarkable Structure Deformation in Phenothiazine Trimer Radical Cation. Org. Lett. 6, 3493–3496 (2004). 15387531 10.1021/ol048698z

[b22] Wu W. . Efficient and stable dye-sensitized solar cells based on phenothiazine sensitizers with thiophene units. J. Mater. Chem. 20, 1772 (2010).

[b23] Kim M. J. . Tuning of spacer groups in organic dyes for efficient inhibition of charge recombination in dye-sensitized solar cells. Dye. Pigment. 95, 134–141 (2012).

[b24] Qiu X. . Synthesis of phenothiazine-functionalized porphyrins with high fluorescent quantum yields. Tetrahedron Lett. 49, 7446–7449 (2008).

[b25] Tian H. . Phenothiazine derivatives for efficient organic dye-sensitized solar cells. Chem. Commun. (Camb.) 36, 3741–3743 (2007). 10.1039/b707485a 17851613

[b26] Liang M. & Chen J. Arylamine organic dyes for dye-sensitized solar cells. Chem. Soc. Rev. 42, 3453–3488 (2013). 23396530 10.1039/c3cs35372a

[b27] Kim S. H. . Effect of Five-Membered Heteroaromatic Linkers to the Performance of Phenothiazine-Based Dye-Sensitized Solar Cells. Org. Lett. 13, 5784–5787 (2011). 21970651 10.1021/ol2023517

[b28] Huang Z.-S., Meier H. & Cao D. Phenothiazine-based dyes for efficient dye-sensitized solar cells. J. Mater. Chem. C 4, 2404–2426 (2016).

[b29] Kim S. H. . The effect of N-substitution and ethylthio substitution on the performance of phenothiazine donors in dye-sensitized solar cells. Dyes Pigments 97, 262–271 (2013).

[b30] Cao D. . Enhanced Performance of the Dye-Sensitized Solar Cells with Phenothiazine-Based Dyes Containing Double D−A Branches. Org. Lett. 13, 1610–1613 (2011). 21370866 10.1021/ol2000167

[b31] Park S. S., Won Y. S., Choi Y. C. & Kim J. H. Molecular Design of Organic Dyes with Double Electron Acceptor for Dye-Sensitized Solar Cell. Energy and Fuels 23, 3732–3736 (2009).

[b32] Huang J. H. & Lee K. C. Highly Stable, Solution-Processable Phenothiazine Derivative as Hole Collection Material for Organic Solar Cells. ACS Appl. Mater. Interfaces 6, 7680–7685 (2014). 24785782 10.1021/am5009503

[b33] Li Z. . Design and synthesis of solution processable small molecules towards high photovoltaic performance. J. Mater. Chem. 21, 2159–2168 (2011).

[b34] Kumar S., Singh M., Jou J.-H. & Ghosh S. Trend breaking substitution pattern of phenothiazine with acceptors as a rational design platform for blue emitters. J. Mater. Chem. C 4, 6769–6777 (2016).

[b35] Ahn Y. . Electroluminescence Characteristics of a New Green-Emitting Phenylphenothiazine Derivative with Phenylbenzimidazole Substituent. Bull. Korean Chem. Soc. 34, 107–111 (2013).

[b36] Fidder H., Knoester J. & Wiersma D. A. Optical properties of disordered molecular aggregates: A numerical study. J. Chem. Phys. 95, 7880–7890 (1991).

[b37] Spano F. C. The Spectral Signatures of Frenkel Polarons in H- and J-Aggregates. Acc. Chem. Res. 43, 429–439 (2010). 20014774 10.1021/ar900233v

[b38] Chang J.-F. . Molecular-weight dependence of interchain polaron delocalization and exciton bandwidth in high-mobility conjugated polymers. Phys. Rev. B 74, 115318 (2006).

[b39] Sun X. . Novel Electroactive and Photoactive Molecular Materials Based on Conjugated Donor−Acceptor Structures for Optoelectronic Device Applications. J. Phys. Chem. B 109, 10786–10792 (2005). 16852311 10.1021/jp0509515

[b40] Sang G., Zou Y. & Li Y. Two Polythiophene Derivatives Containing Phenothiazine Units: Synthesis and Photovoltaic Properties. J. Phys. Chem. C 112, 12058–12064 (2008).

[b41] Siu C. H. . Synthesis and Characterization of Phenothiazine-Based Platinum(II)–Acetylide Photosensitizers for Efficient Dye-Sensitized Solar Cells. Chem. - A Eur. J. 22, 3750–3757 (2016). 10.1002/chem.201503828 26660631

[b42] Blanco G. D. . Syntheses, Charge Separation, and Inverted Bulk Heterojunction Solar Cell Application of Phenothiazine–Fullerene Dyads. ACS Appl. Mater. Interfaces 8, 8481–8490 (2016). 26990247 10.1021/acsami.6b00561

[b43] Tan Q. . Application of Small Molecule Donor Materials Based on Phenothiazine Core Unit in Bulk Heterojunction Solar Cells. J. Phys. Chem. C 118, 16851–16855 (2014).

[b44] Matsunaga Y. Some new organic semiconductors : thiazineiodine complexes. Helv. Phys. Acta 36, 800–802 (1963).

[b45] Matsunaga Y. Infrared spectra in the characterization of some molecular complexes of the dative type. J. Chem. Phys. 41, 1609–1613 (1964).

[b46] Kan K. & Matsunaga Y. The Electrical Properties of Iodine Complexes of N-Methyl-and N-Ethyl-phenothiazines and Their Mixtures. Bull. Chem. Soc. Jpn. 45, 2096–2100 (1972).

[b47] Matsunaga Y. & Suzuki Y. Electrical and Optical Properties of the Iodine Complexes of Phenoxazine, Phenoselenazine, and Benzophenothiazines. Bull. Chem. Soc. Jpn. 45, 3375–3379 (1972).

[b48] Matsunaga Y. & Suzuki Y. The Cation-radical Salts Derived from Benzo- and Dibenzo-phenothiazines. Bull. Chem. Soc. Jpn. 46, 719–722 (1973).

[b49] Matsunaga Y. Energy and Charge Transfer in Organic Semiconductors, Springer: US: Boston, MA,, (1974).

[b50] Doi S., Inabe T. & Matsunaga Y. Electrical Properties and Constitution of the Phenothiazine–Iodine and Related Complexes. Bull. Chem. Soc. Jpn. 50, 837–841 (1977).

[b51] Bakulin A. A. . The role of driving energy and delocalized States for charge separation in organic semiconductors. Science 335, 1340–1344 (2012). 22362882 10.1126/science.1217745

[b52] Shoaee S. . Acceptor Energy Level Control of Charge Photogeneration in Organic Donor/Acceptor Blends. J. Am. Chem. Soc. 132, 12919–12926 (2010). 20804126 10.1021/ja1042726

[b53] Chang Y. J. . Organic dyes containing oligo-phenothiazine for dye-sensitized solar cells. J. Mater. Chem. 22, 21704–21712 (2012).

[b54] Iqbal Z. . Phenothiazine-based dyes with bilateral extension of π-conjugation for efficient dye-sensitized solar cells. Dyes Pigments 96, 722–731 (2013).

[b55] Hua Y. . Co-sensitization of 3D bulky phenothiazine-cored photosensitizers with planar squaraine dyes for efficient dye-sensitized solar cells. J. Mater. Chem. A 3, 13848–13855 (2015).

[b56] Lin R. Y.-Y. . High-Performance Aqueous/Organic Dye-Sensitized Solar Cells Based on Sensitizers Containing Triethylene Oxide Methyl Ether. ChemSusChem 8, 2503–2513 (2015). 26098636 10.1002/cssc.201500589

[b57] Bejan A., Shova S., Damaceanu M.-D., Simionescu B. C. & Marin L. Structure-Directed Functional Properties of Phenothiazine Brominated Dyes: Morphology and Photophysical and Electrochemical Properties. Cryst. Growth Des. 16, 3716–3730 (2016).

[b58] Würthner F., Kaiser T. E. & Saha-Möller C. R. J-Aggregates: From Serendipitous Discovery to Supramolecular Engineering of Functional Dye Materials. Angew. Chemie - Int. Ed. 50, 3376–3410 (2011). 10.1002/anie.201002307 21442690

[b59] Heeger A. J. 25th Anniversary Article: Bulk Heterojunction Solar Cells: Understanding the Mechanism of Operation. Adv. Mater. 26, 10–28 (2014). 24311015 10.1002/adma.201304373

[b60] Macdonald J. R. & Kenan W. R. Impedance Spectroscopy: Emphasizing Solid Materials and Systems, Wiley-Interscience: New York, (1987).

[b61] Tanase C., Meijer E. J., Blom P. W. M. & De Leeuw D. M. Unification of the Hole Transport in Polymeric Field-Effect Transistors and Light-Emitting Diodes. Phys. Rev. Lett. 91, 216601 (2003). 14683323 10.1103/PhysRevLett.91.216601

[b62] Kassing R. Calculation of the frequency dependence of the admittance of SCLC diodes. Phys. status solidi 28, 107–117 (1975).

[b63] Tripathi D. C., Tripathi A. K. & Mohapatra Y. N. Mobility determination using frequency dependence of imaginary part of impedance (Im Z) for organic and polymeric thin films. Appl. Phys. Lett. 98, 033304 (2011).

[b64] Frenkel J. On Pre-Breakdown Phenomena in Insulators and Electronic Semi-Conductors. Phys. Rev. 54, 647–648 (1938).

[b65] Poole H. H. On the dielectric constant and electrical conductivity of mica in intense fields. Philos. Mag. Ser. 32, 112–129 (1916).

[b66] Dou J. H. . Systematic Investigation of Side-Chain Branching Position Effect on Electron Carrier Mobility in Conjugated Polymers. Adv. Funct. Mater. 24, 6270–6278 (2014).

[b67] Ma Z., Geng H., Wang D. & Shuai Z. Influence of alkyl side-chain length on the carrier mobility in organic semiconductors: herringbone vs. pi–pi stacking. J. Mater. Chem. C 4, 4546–4555 (2016).

[b68] Anthony J. E. Functionalized Acenes and Heteroacenes for Organic Electronics. Chem. Rev. 106, 5028–5048 (2006). 17165682 10.1021/cr050966z

[b69] Laurinaviciute R., Ostrauskaite J., Skuodis E., Grazulevicius G. V. & Jankauskas V. Synth. Met. 192, 50–55 (2014).

[b70] Cheng Y.-J. . A phenothiazine/dimesitylborane hybrid material as a bipolar transport host of red phosphor. J. Mater. Chem. C 4, 9499–9508 (2016).

[b71] D’Andrade B. W. . Relationship between the ionization and oxidation potentials of molecular organic semiconductors. Org. Electron. physics, Mater. Appl. 6, 11–20 (2005).

[b72] Parr R. G. & Yang W. Density-functional theory of atoms and molecules, Oxford University Press, New York, (1989).

[b73] Casida M. E. Recent Advances in Density Functional Methods, ed. Chong D. P., World Scientific: Singapore (1995).

