# Supplementary material for: Crystallisation-enhanced bulk hole mobility in phenothiazine-based organic semiconductors
Source: Sci Rep. 2017 Apr 12;7:46268. doi: 10.1038/srep46268 (PMC5388875; doi:10.1038/srep46268)
Supplement: Supplementary Information [file srep46268-s1.pdf]

Supplementary Information (SI)

Crystallisation-enhanced bulk hole mobility in phenothiazines-based organic semiconductors

Durgaprasad Shinde,[a] Jagadish Salunke,*[b] N. R. Candeias,[b] Francesca Tinti,[c] Massimo Gazzano,[c] Prakash Wadgaonkar,[a] Arri Priimägi,[b] Nadia Camaioni,*[c] Paola Vivo*[b]

[a.] *Polymer Science and Engineering Division, CSIR-National Chemical Laboratory, Dr Homi Bhabha Road, Pune 411008, India*
[b.] *Laboratory of Chemistry and Bioengineering, Tampere University of Technology, P.O. Box 541, FI-33101 Tampere, Finland.*
[c.] *Istituto per la Sintesi Organica e la Fotoreattività, Consiglio Nazionale delle Ricerche, via P. Gobetti 101, I-40129 Bologna, Italy*

Table of contents

- **Figure S1.** ^{1}H and ^{13}C NMR of 10-(4-methoxyphenyl)-10H-phenothiazine **1**.
- **Figure S2.** ^{1}H and ^{13}C NMR of 10-(4-methoxyphenyl)-10H-phenothiazine-3-carbaldehyde **2**.
- **Figure S3.** ^{1}H and ^{13}C NMR of 2-((10-(4-methoxyphenyl)-10H-phenothiazin-3-yl) methylene) Malononitrile (**O-1**).
- **Figure S4.** ^{1}H and ^{13}C NMR of 7-bromo-10-(4-methoxyphenyl)-10H-phenothiazine-3-carbaldehyde **3**.
- **Figure S5.** ^{1}H and ^{13}C NMR of 7,10-bis(4-methoxyphenyl)-10H-phenothiazine-3-carbaldehyde **4.**
- **Figure S6.** ^{1}H and ^{13}C NMR of 2-((7,10-bis(4-methoxyphenyl)-10H-phenothiazin-3-yl) methylene) Malononitrile(**O-2**).
- **Figure S7.** ^{1}H and ^{13}C NMR of 2-bromo-6-butoxynaphthalene **6**.
- **Figure S8.** ^{1}H and ^{13}C NMR of 2-(6-butoxynaphthalen-2-yl)-4,4,5,5-tetramethyl-1,3,2-Dioxaborolane **7**.
- **Figure S9.** ^{1}H and ^{13}C NMR of 7-(6-butoxynaphthalen-2-yl)-10-(4-methoxyphenyl)-10H-phenothiazine-3-carbaldehyde **5.**
- **Figure S10.** ^{1}H and ^{13}C NMR of 2-((7-(6-butoxynaphthalen-2-yl)-10-(4-methoxyphenyl)-10H-phenothiazin-3-yl) methylene) malononitrile (**O-3**).
- **Figure S11.** MALDI-TOF spectra of **O-1**, **O-2**, and **O-3**.
- **Figure S12.** DPV voltammograms of **O-1**, **O-2** and **O-3**.
- **Figure S13.** (a) DSC data for **O-1**, **O-2** and **O-3**; (b) TGA data for **O-1**, **O-2** and **O-3**.
- **Figure S14.** XRD patterns of powder samples (p) and of film samples (f) as raw data.
- **Details on synthesis**
- **Computational analysis**
- **Atomic coordinates for all the optimized species**
- **References**

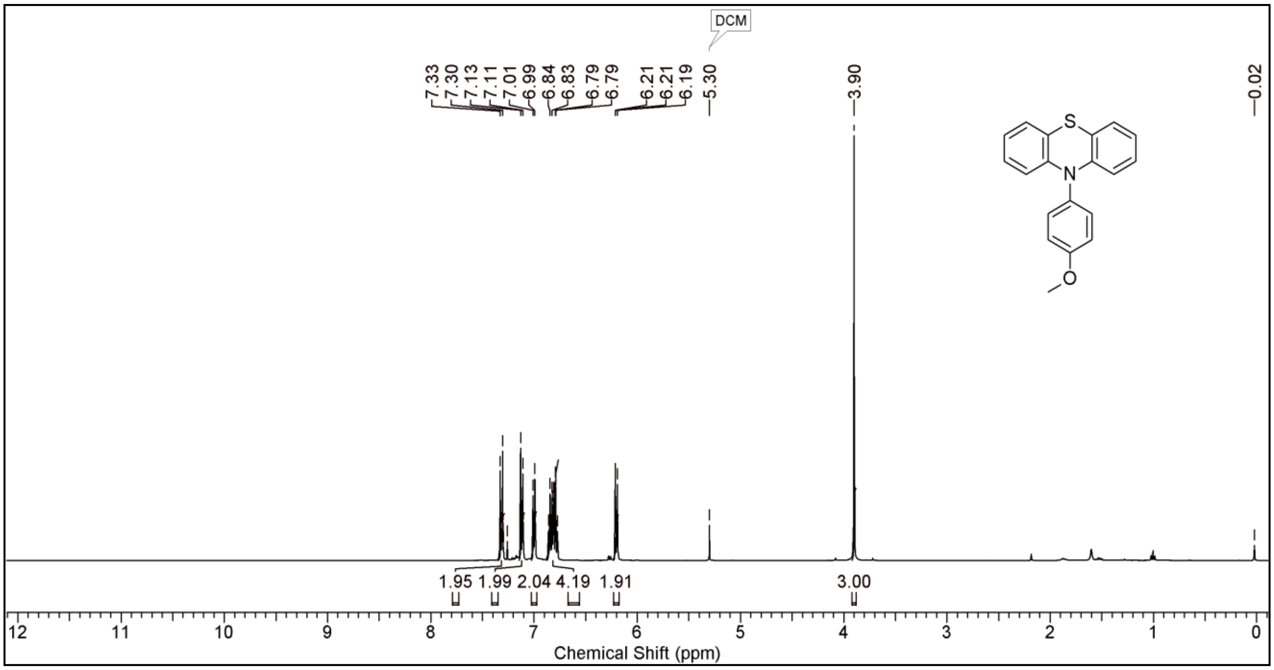

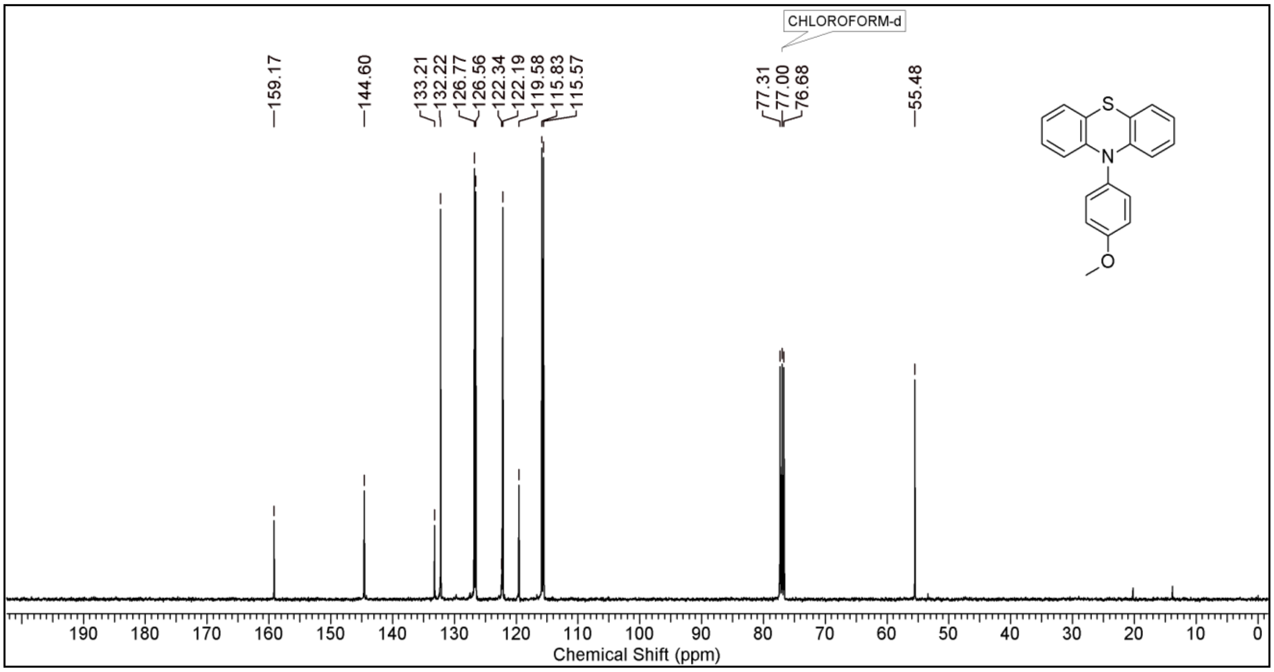

Figure S1. ^{1}H and ^{13}C NMR of 10-(4-methoxyphenyl)-10H-phenothiazine **1**.

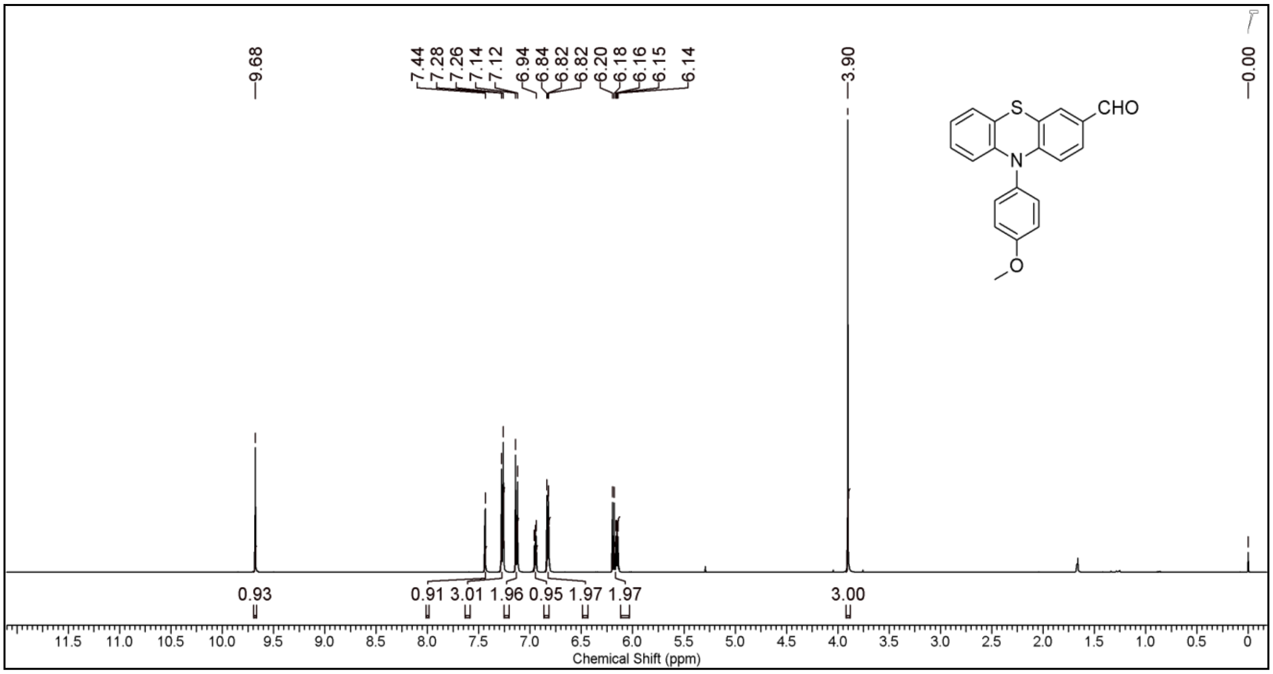

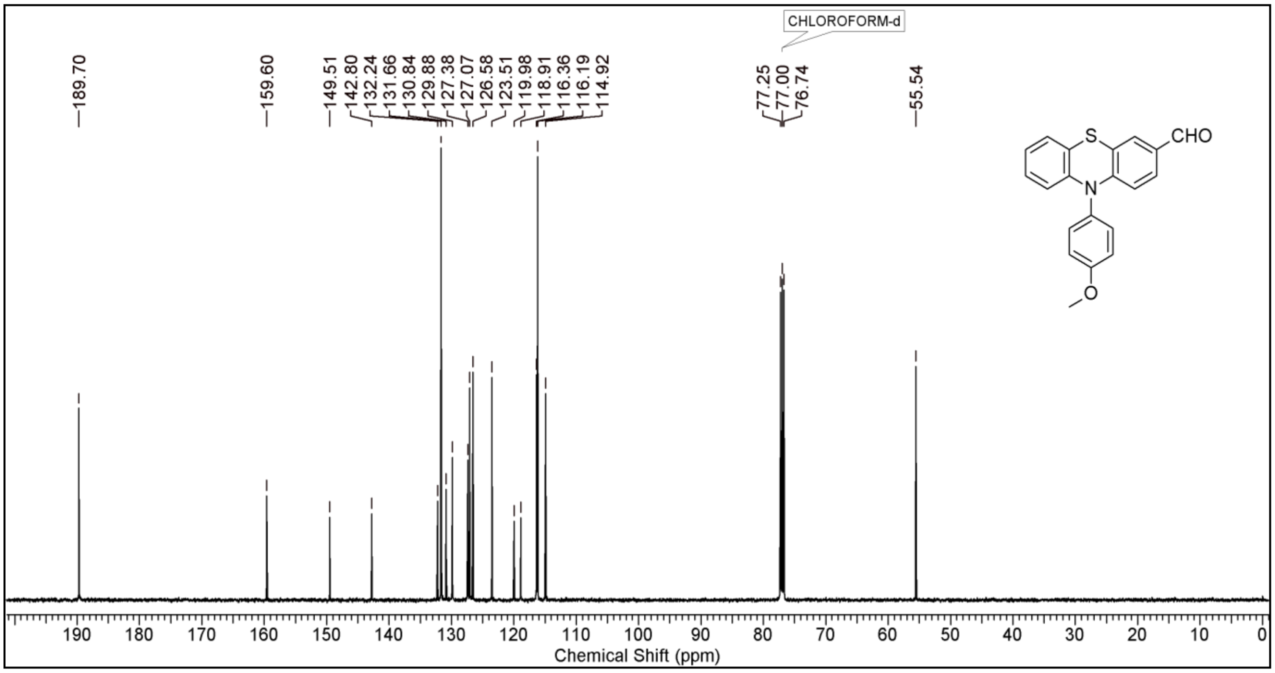

Figure S2. ^{1}H and ^{13}C NMR of 10-(4-methoxyphenyl)-10H-phenothiazine-3-carbaldehyde **2**.

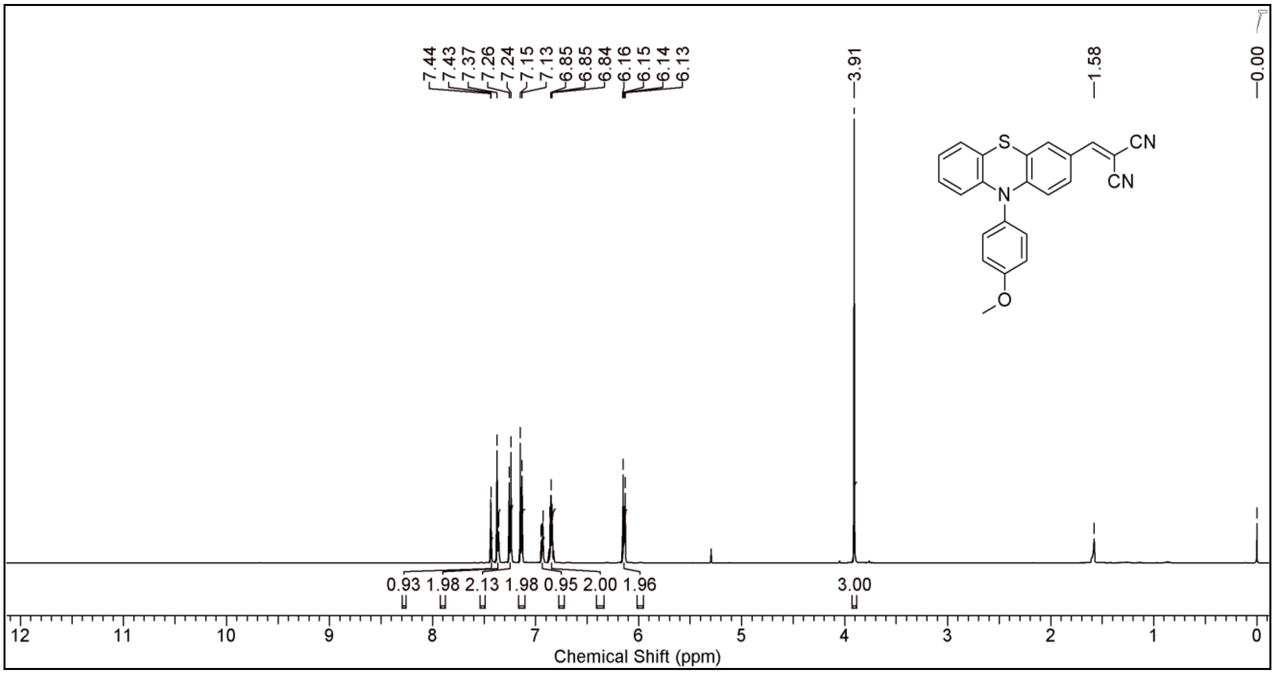

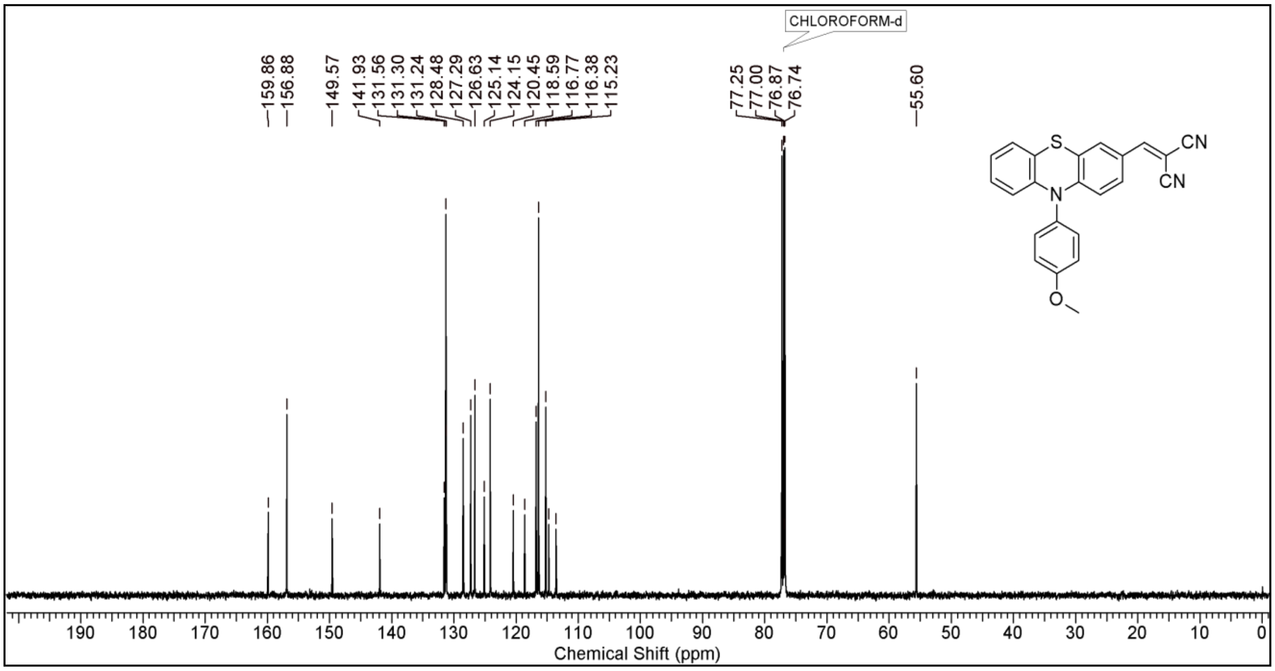

Figure S3. ^{1}H and ^{13}C NMR of 2-((10-(4-methoxyphenyl)-10H-phenothiazin-3-yl) methylene) Malononitrile (**O-1**).

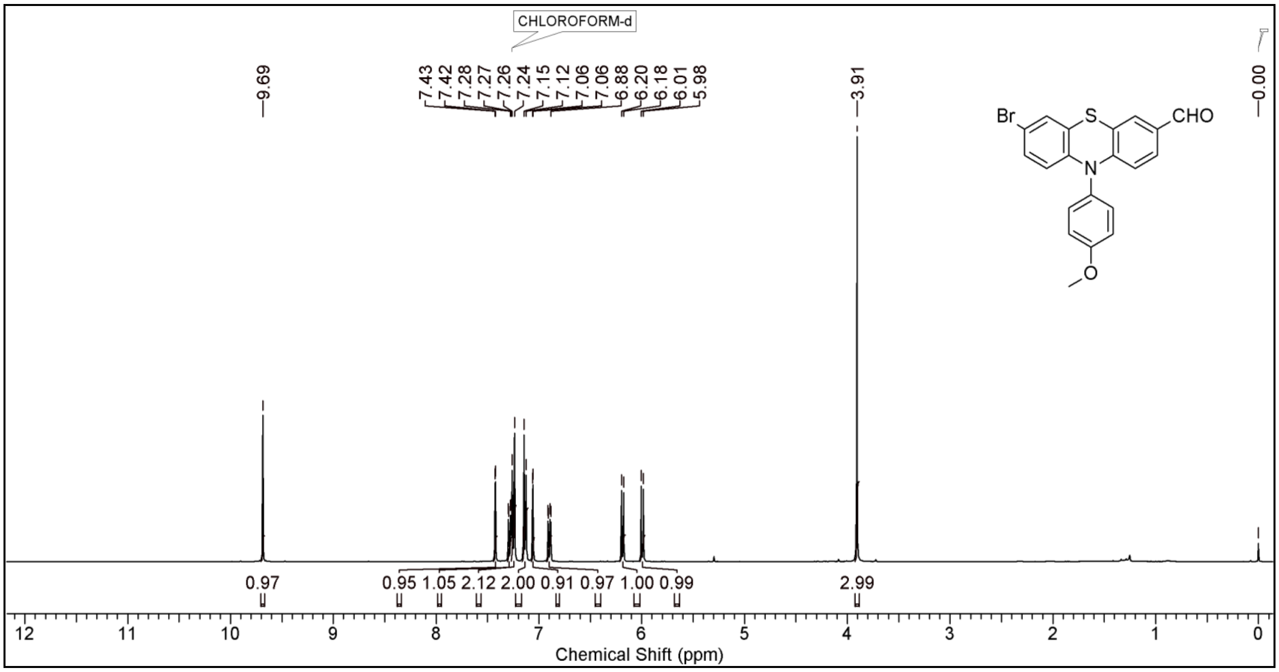

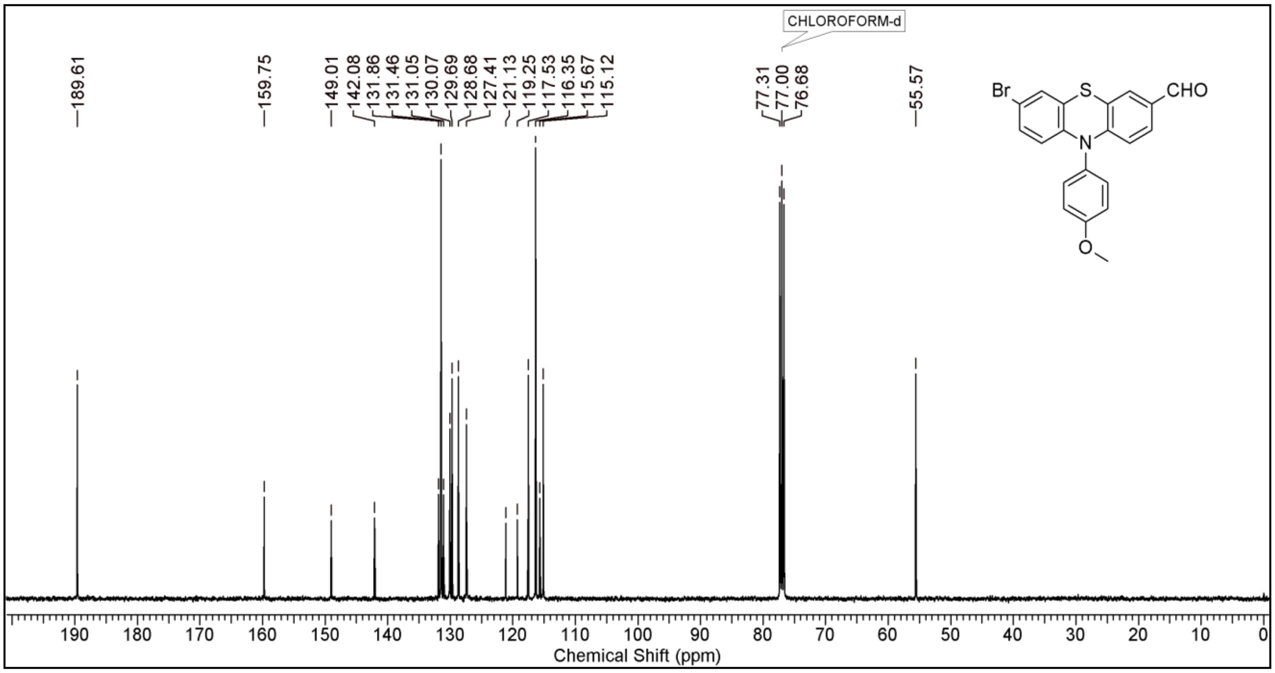

Figure S4. ^{1}H and ^{13}C NMR of 7-bromo-10-(4-methoxyphenyl)-10H-phenothiazine-3-carbaldehyde **3**.

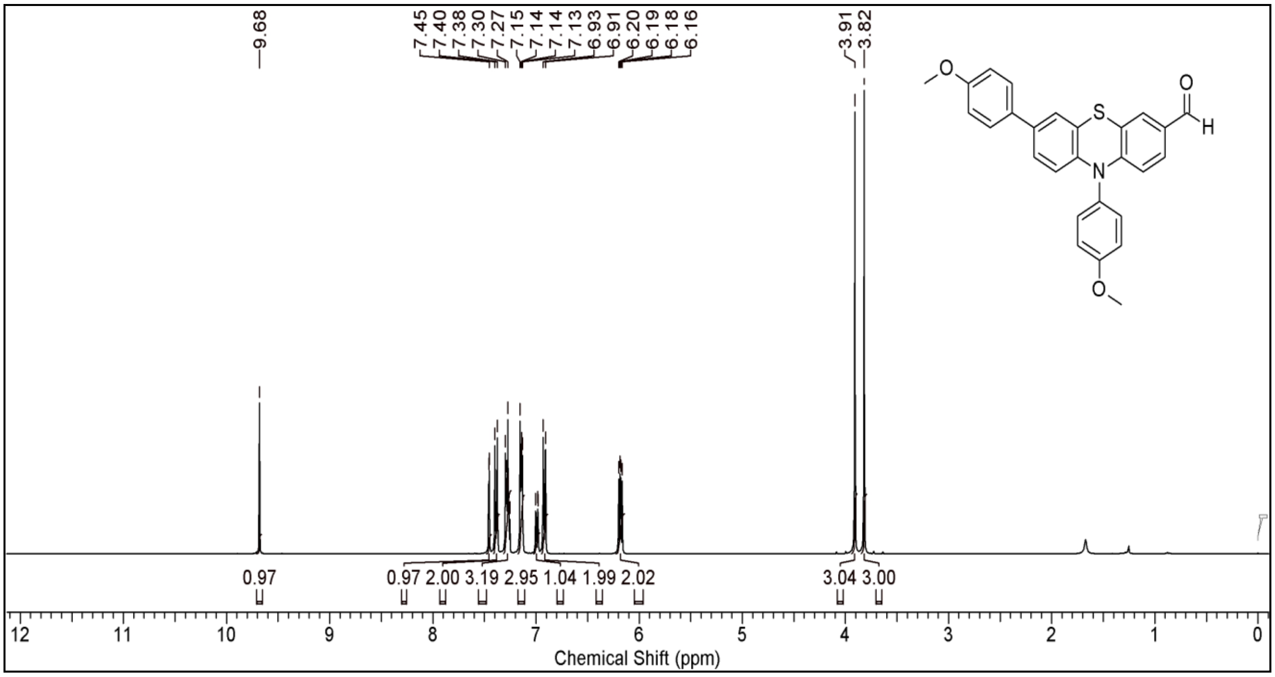

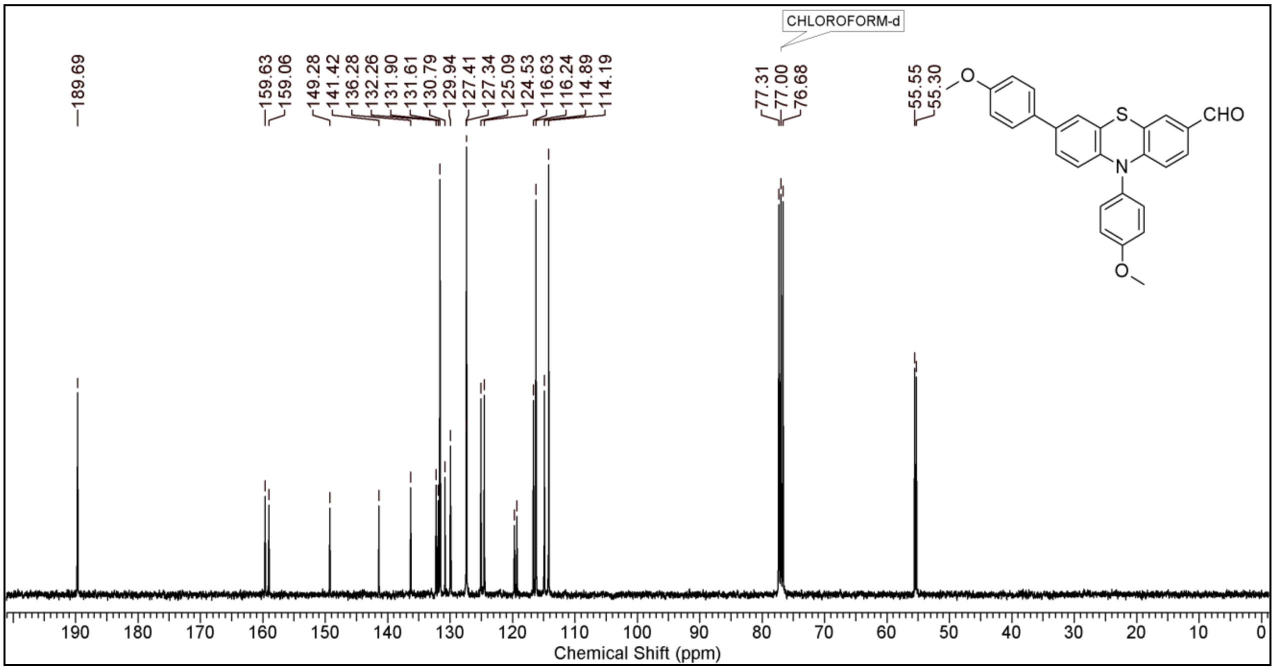

Figure S5. ^{1}H and ^{13}C NMR of 7,10-bis(4-methoxyphenyl)-10H-phenothiazine-3-carbaldehyde **4.**

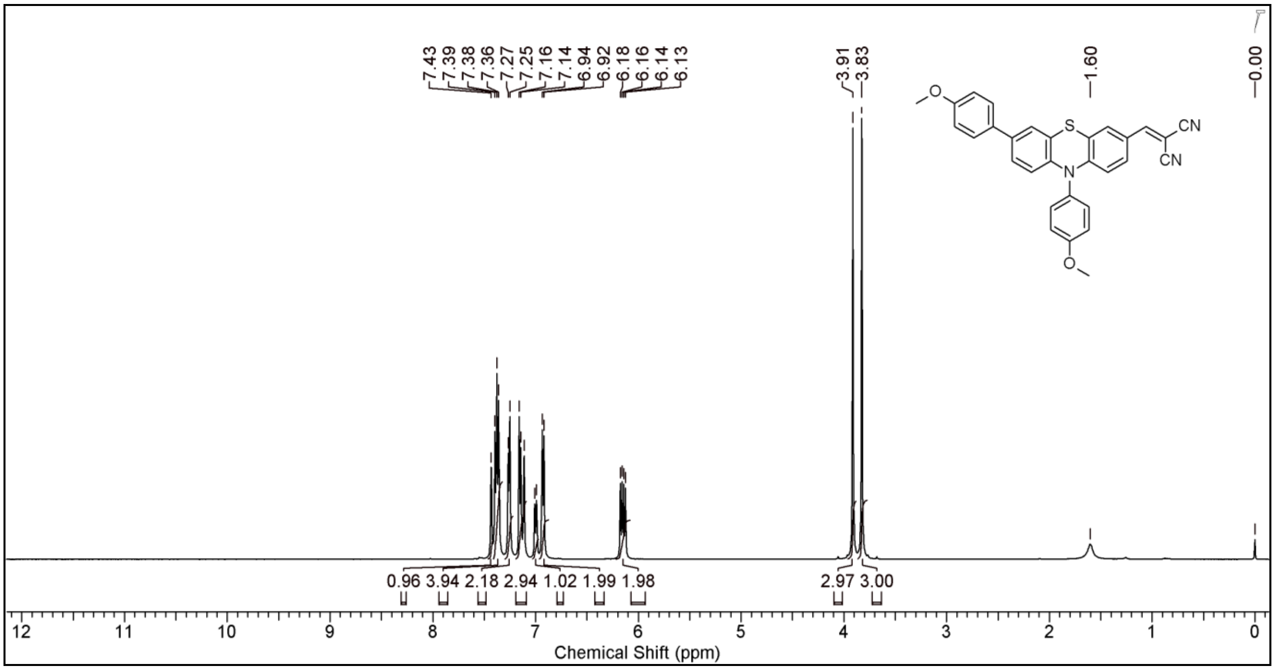

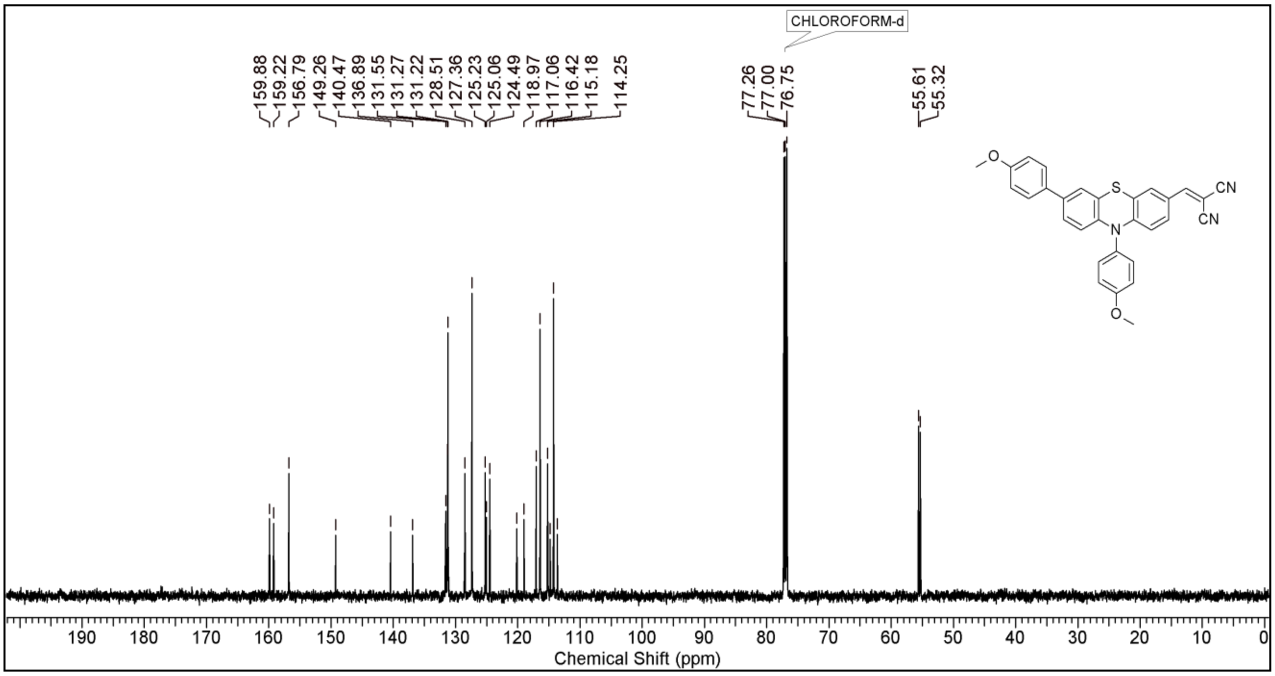

Figure S6. ^{1}H and ^{13}C NMR of 2-((7,10-bis(4-methoxyphenyl)-10H-phenothiazin-3-yl) methylene) Malononitrile(**O-2**).

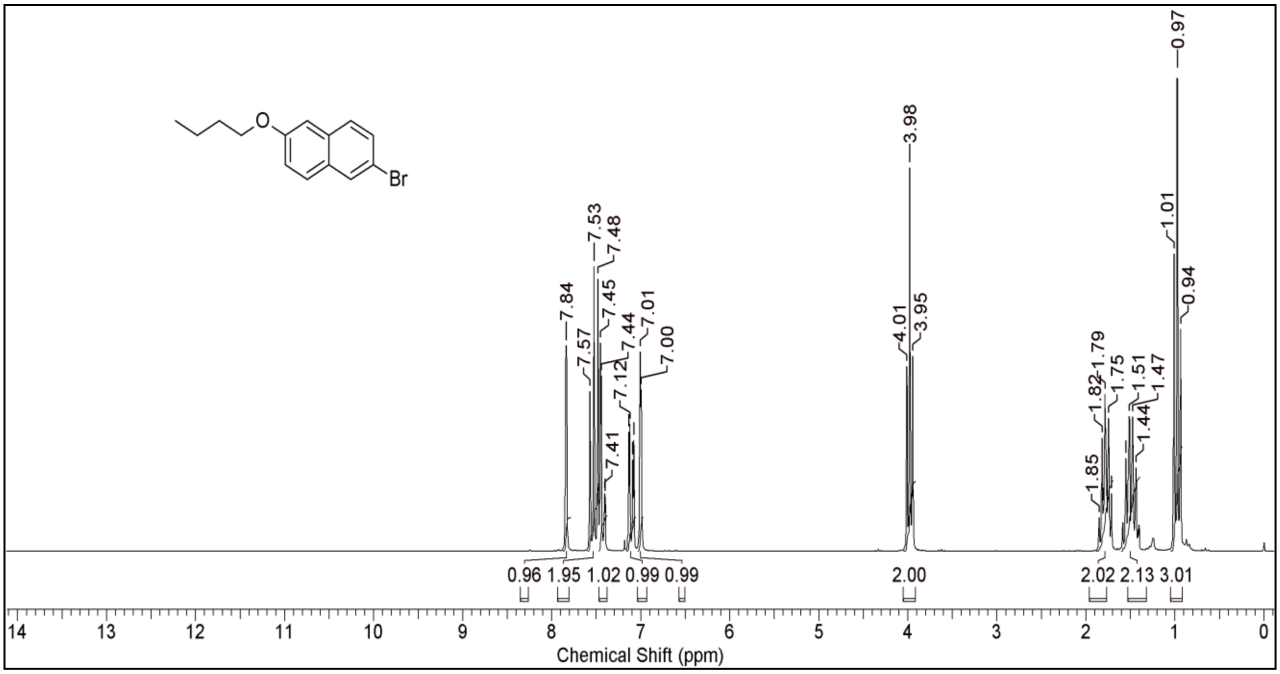

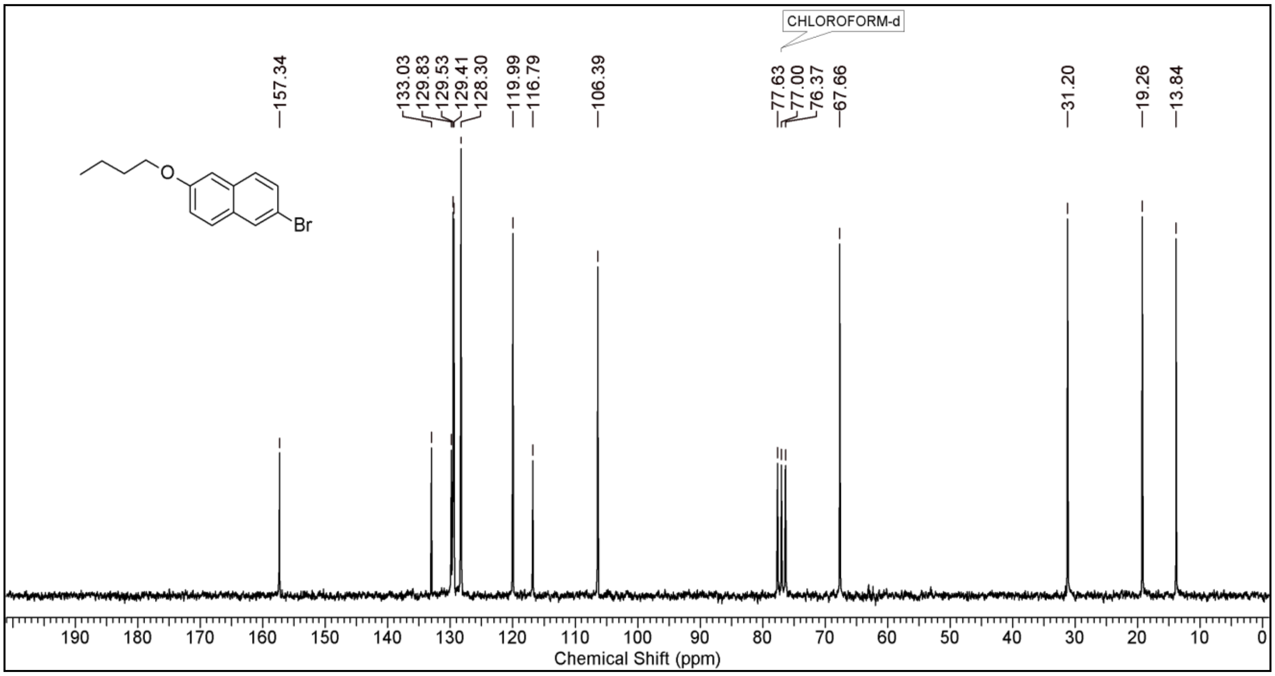

Figure S7. ^{1}H and ^{13}C NMR of 2-bromo-6-butoxynaphthalene **6**.

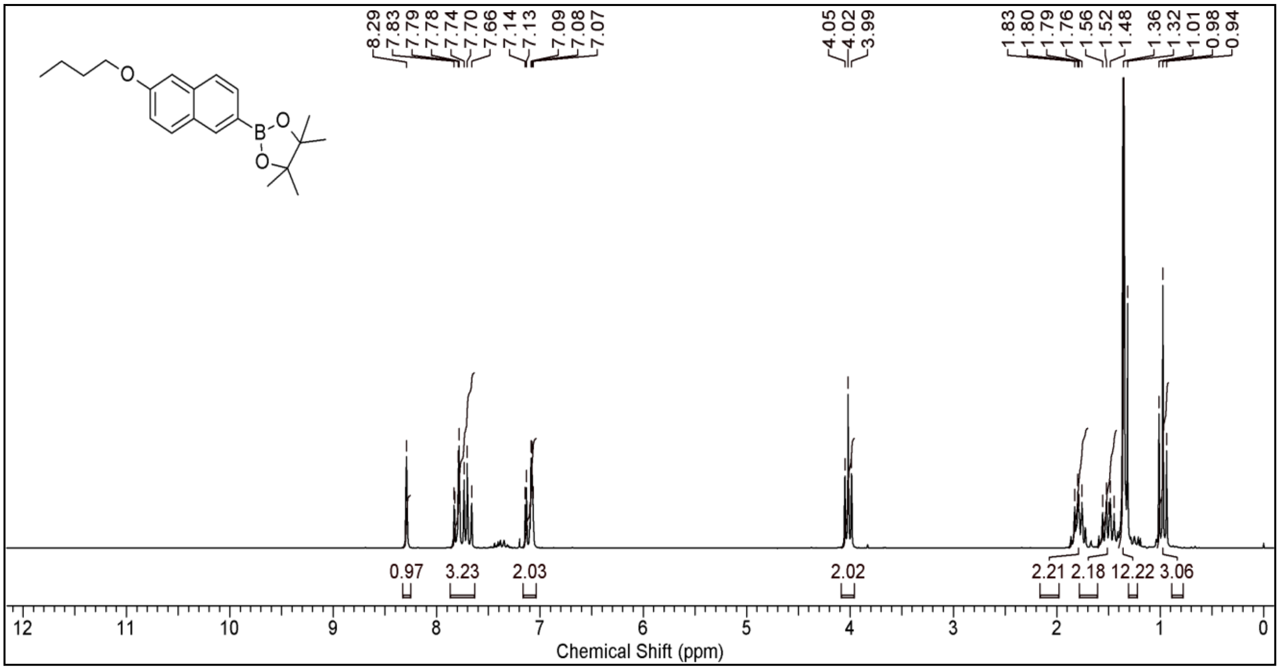

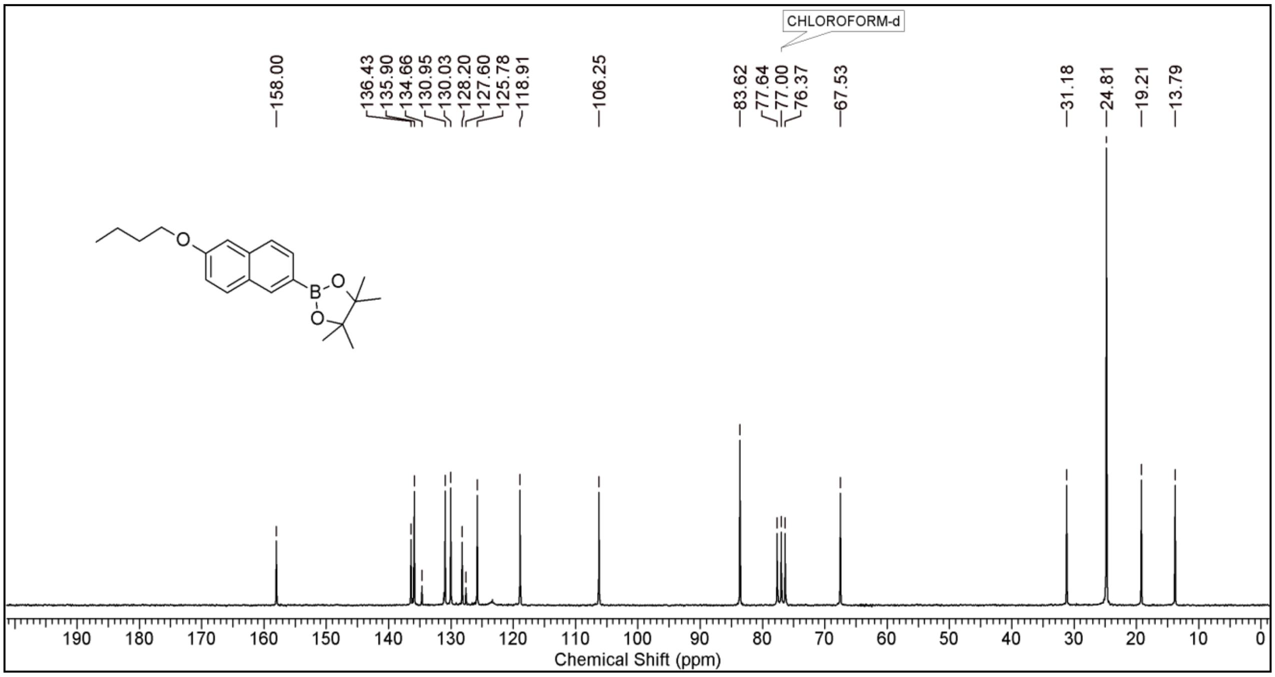

Figure S8. ^{1}H and ^{13}C NMR of 2-(6-butoxynaphthalen-2-yl)-4,4,5,5-tetramethyl-1,3,2-Dioxaborolane **7**.

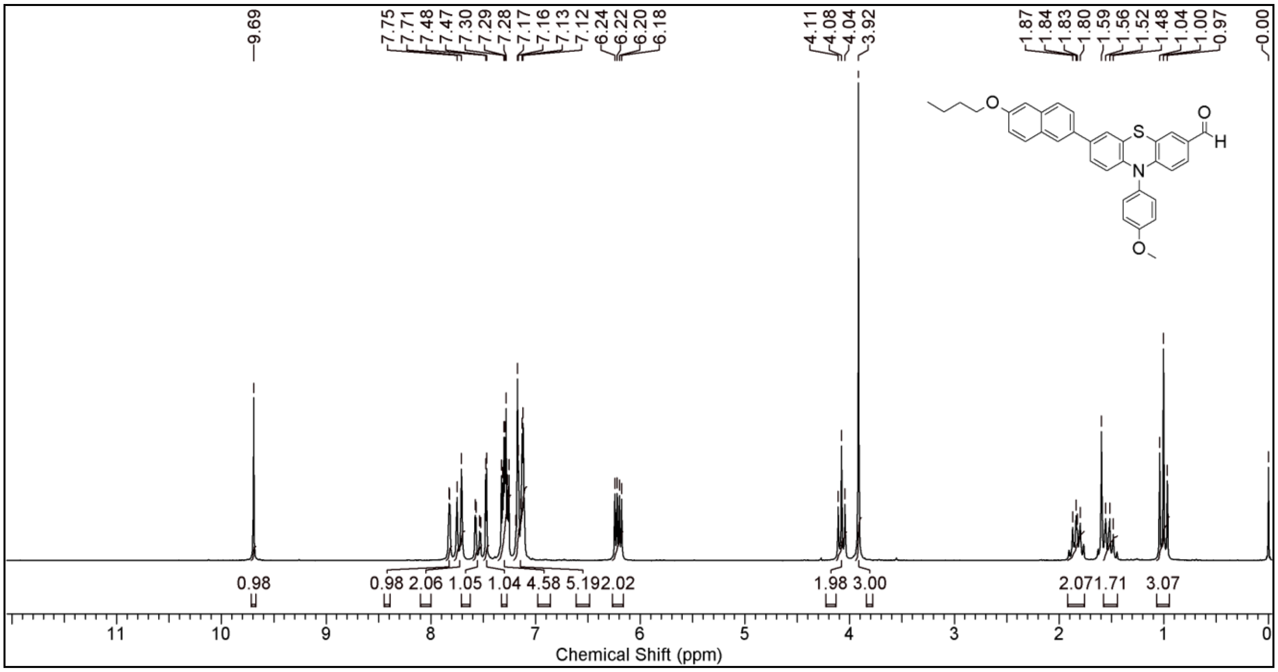

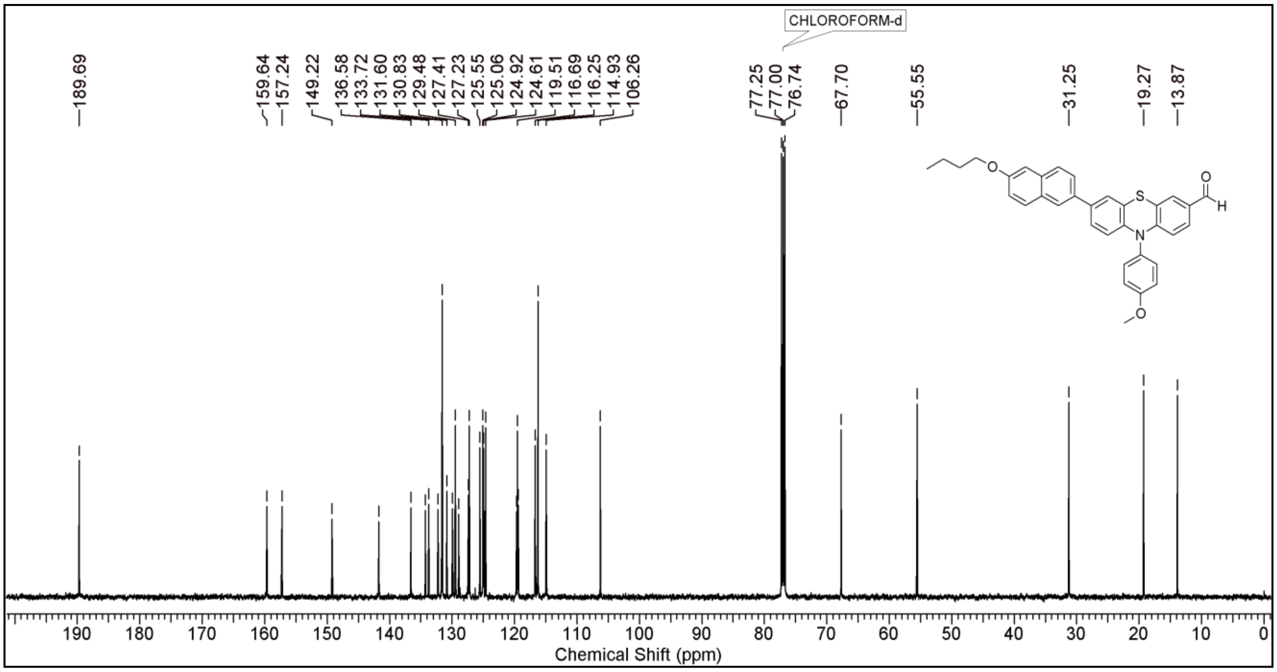

Figure S9. ^{1}H and ^{13}C NMR of 7-(6-butoxynaphthalen-2-yl)-10-(4-methoxyphenyl)-10H-phenothiazine-3-carbaldehyde **5.**

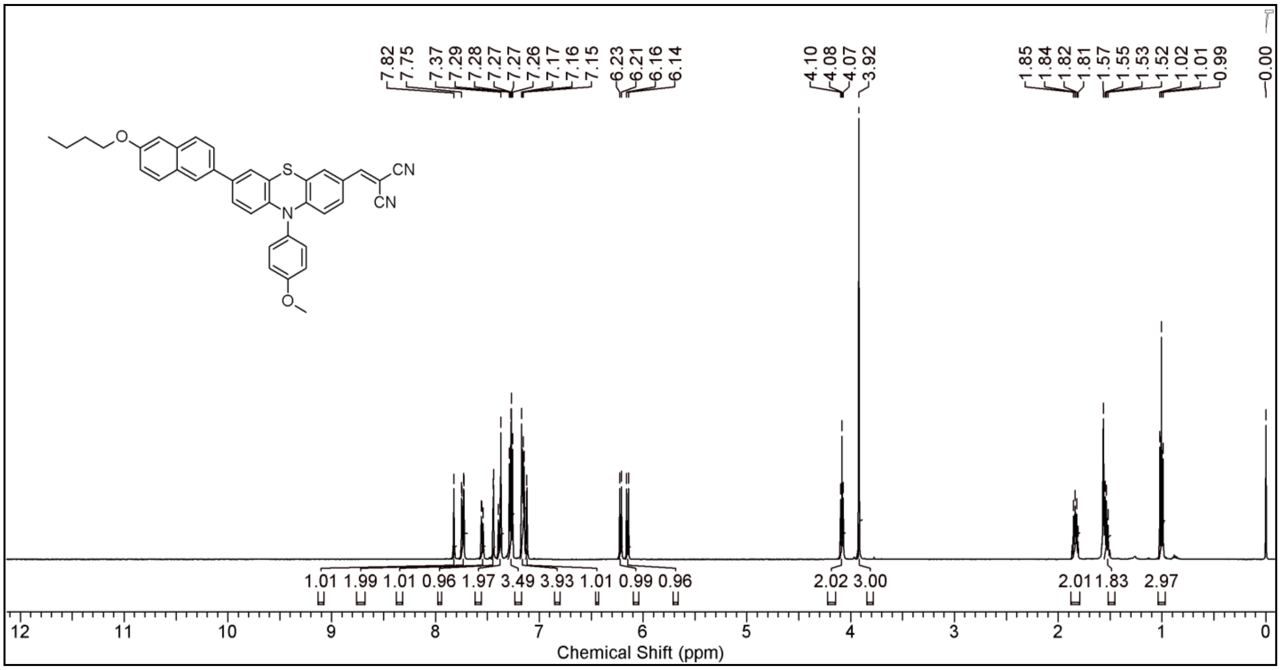

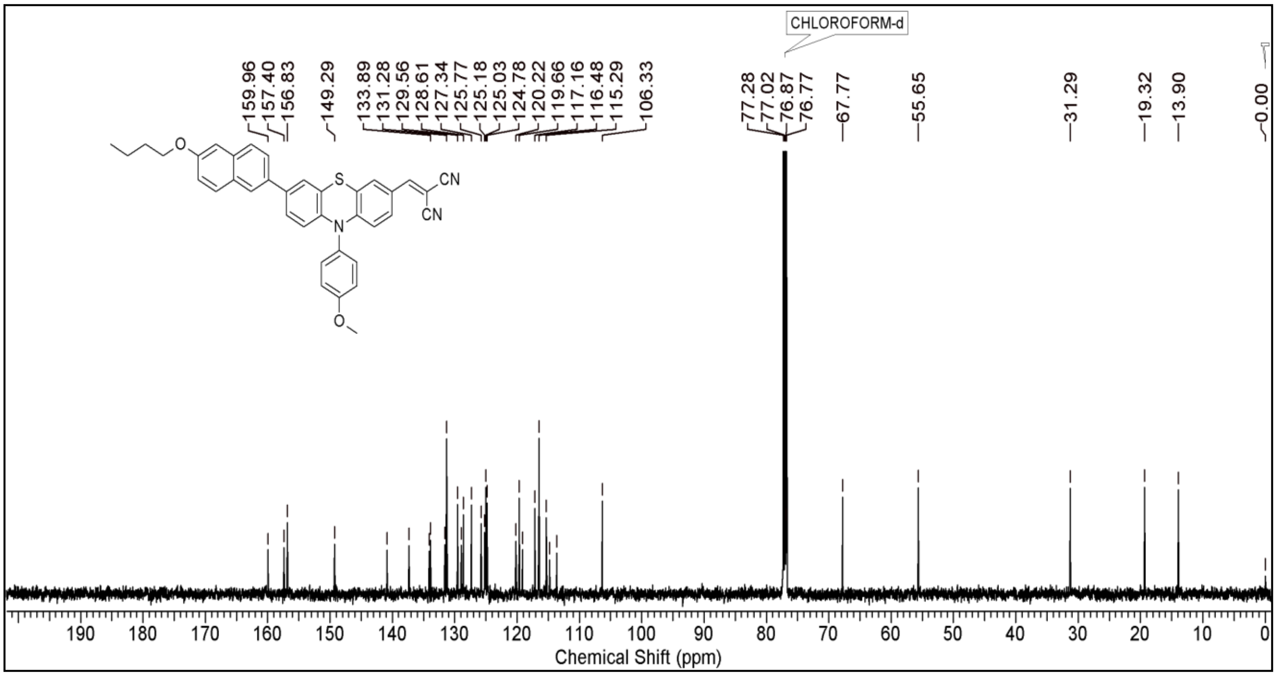

Figure S10. ^{1}H and ^{13}C NMR of 2-((7-(6-butoxynaphthalen-2-yl)-10-(4-methoxyphenyl)-10H-phenothiazin-3-yl) methylene) malononitrile (**O-3**).

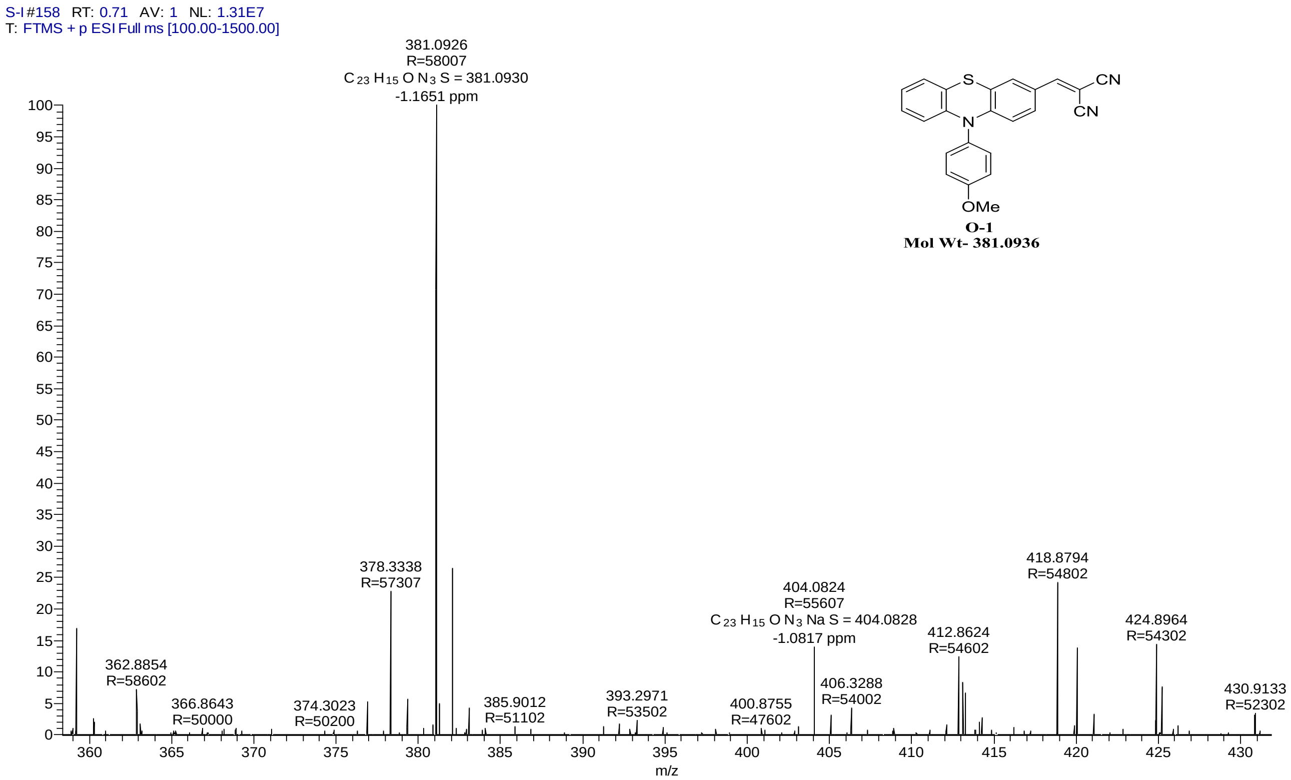
S-I #158 RT: 0.71 AV: 1 NL: 1.31E7
T: FTMS + p ESI Full ms [100.00-1500.00]
381.0926
R=58007
C 23 H 15 O N 3 S = 381.0930
-1.1651 ppm
378.3338
R=57307
362.8854
R=58602
366.8643
R=50000
374.3023
R=50200
385.9012
R=51102
393.2971
R=53502
400.8755
R=47602
404.0824
R=55607
C 23 H 15 O N 3 Na S = 404.0828
-1.0817 ppm
406.3288
R=54002
412.8624
R=54602
418.8794
R=54802
424.8964
R=54302
430.9133
R=52302
m/z
S
N
CN
CN
OMe
O-1
Mol Wt- 381.0936

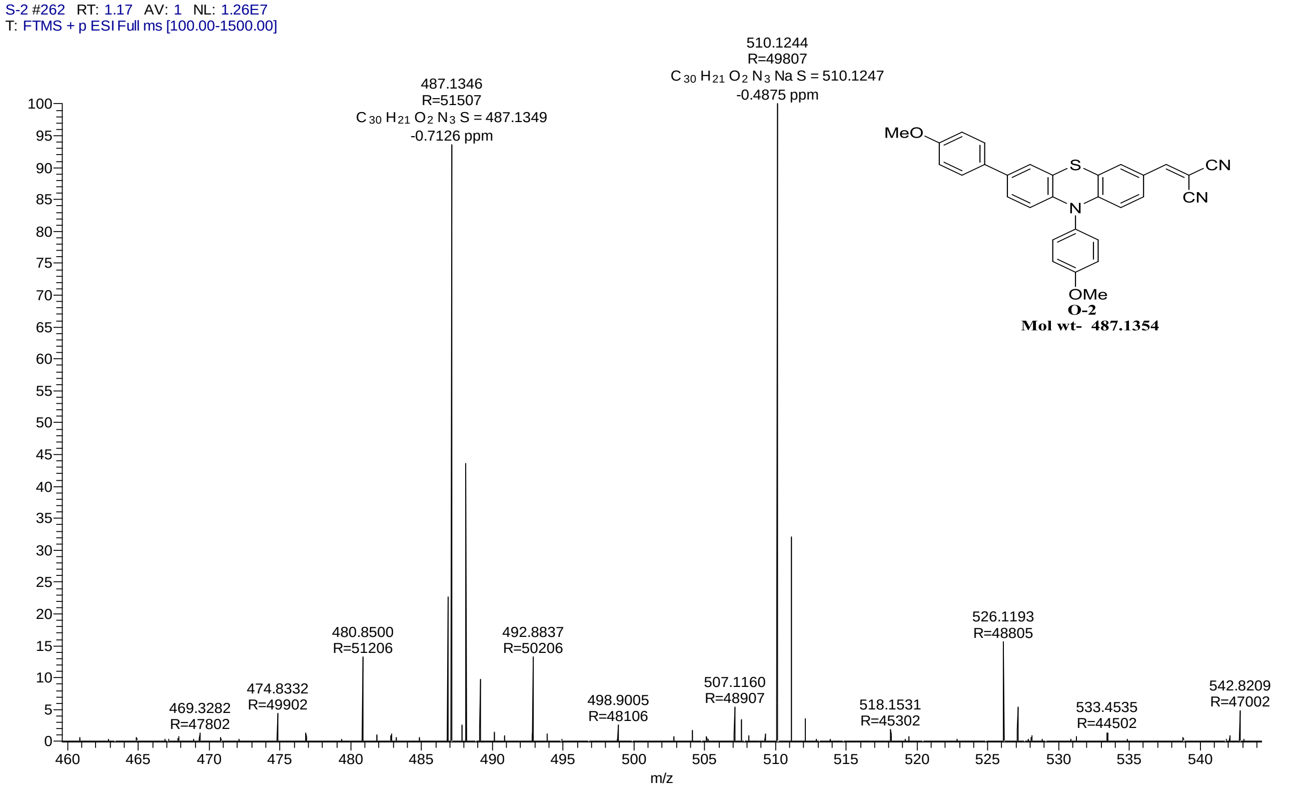
S-2 #262 RT: 1.17 AV: 1 NL: 1.26E7
T: FTMS + p ESI Full ms [100.00-1500.00]
510.1244
R=49807
C 30 H 21 O 2 N 3 Na S = 510.1247
-0.4875 ppm
487.1346
R=51507
C 30 H 21 O 2 N 3 S = 487.1349
-0.7126 ppm
469.3282
R=47802
474.8332
R=49902
480.8500
R=51206
492.8837
R=50206
498.9005
R=48106
507.1160
R=48907
518.1531
R=45302
526.1193
R=48805
533.4535
R=44502
542.8209
R=47002
m/z
MeO
S
N
CN
CN
OMe
O-2
Mol wt- 487.1354

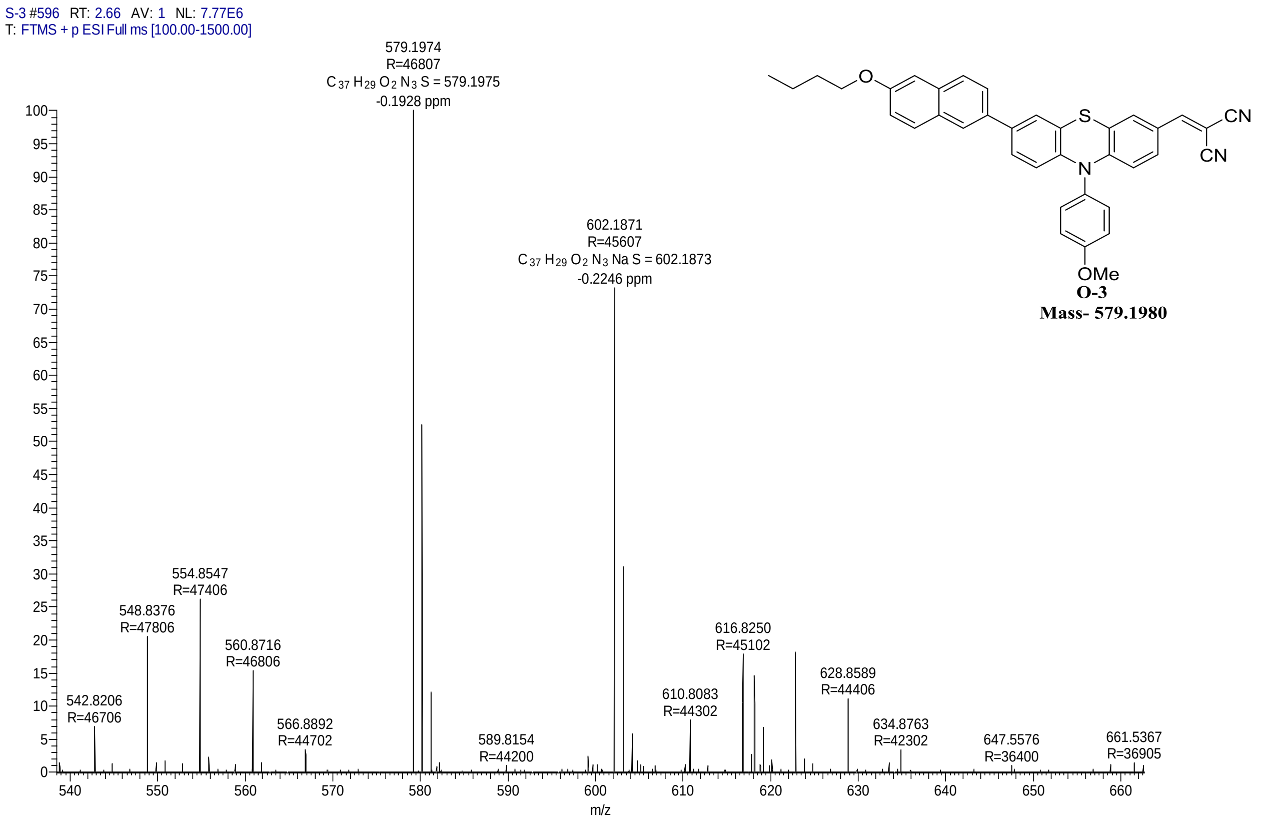

Figure S11. MALDI-TOF spectra of **O-1**, **O-2**, and **O-3**.

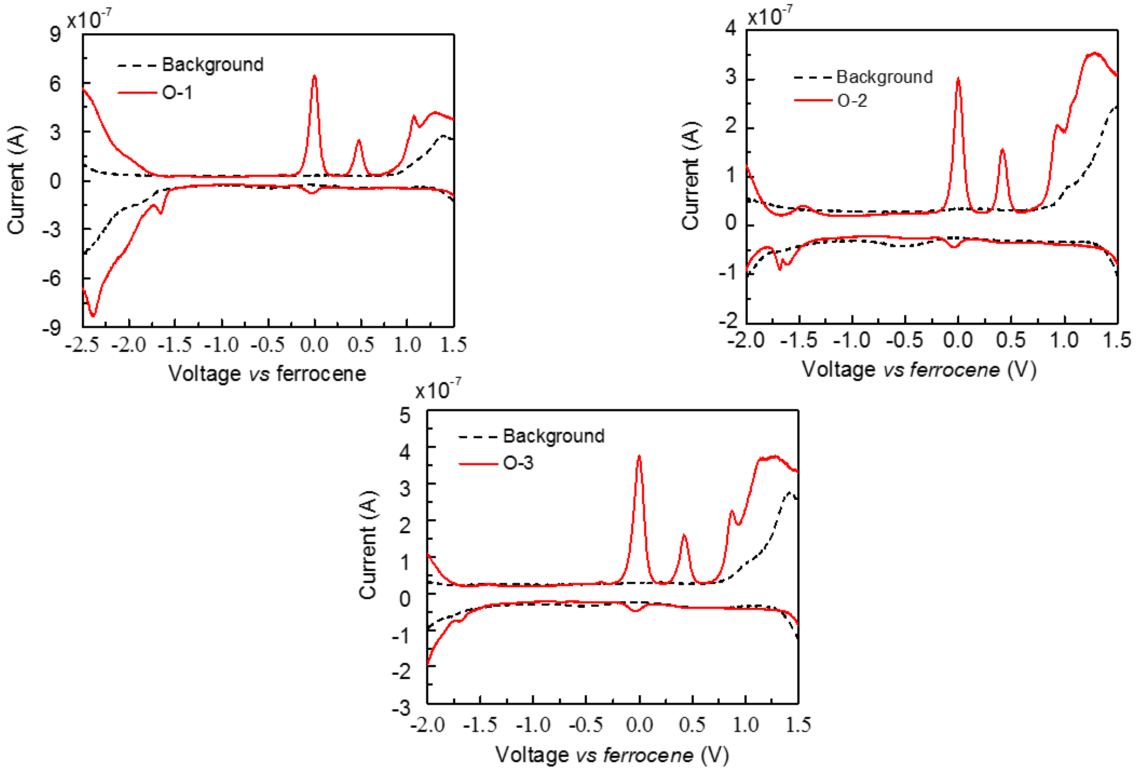

Figure S12. DPV voltammograms of **O-1**, **O-2** and **O-3**.

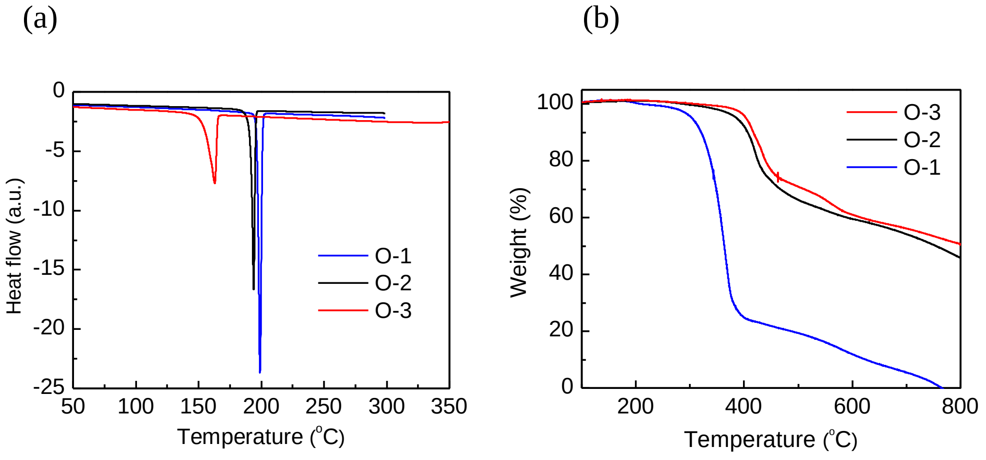

Figure S13. (a) DSC data for **O-1**, **O-2** and **O-3**; (b) TGA data for **O-1**, **O-2** and **O-3**.

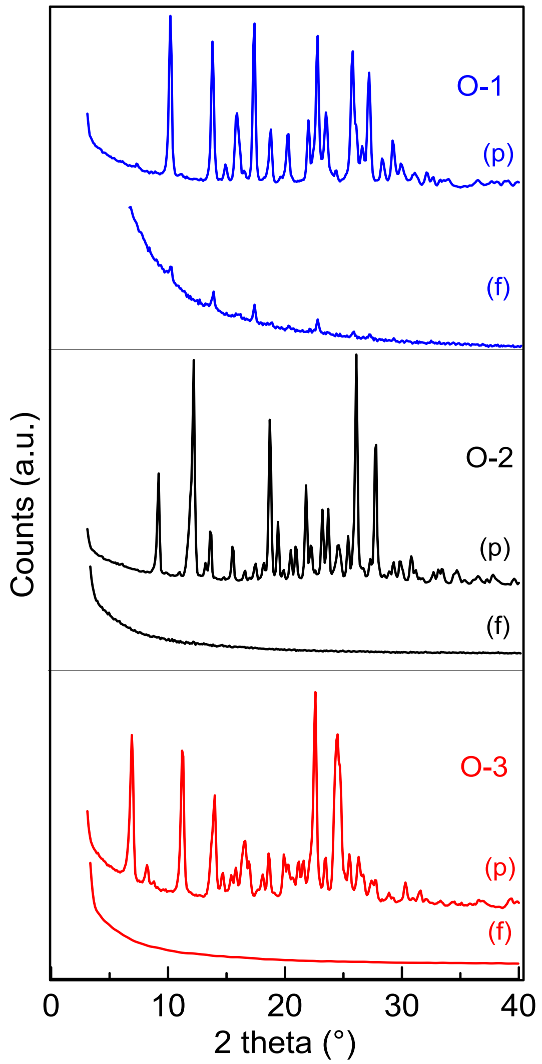

Figure S14. XRD patterns of powder samples (p) and of film samples (f) as raw data.

Details on synthesis

Synthesis of 2-bromo-6-butoxynaphthalene (6). 6-bromo-2-napthol (4 g, 18 mmol), potassium hydroxide (1.2 g, 21 mmol) and dimethyl sulfoxide (50 mL) were added into a 250 mL two necked round bottom flask, and stirred for 30 min. Then, 1-bromo butane (2.3 mL, 21 mmol) was added drop wise to the mixture and reaction mixture was stirred overnight at room temperature. After completion of the reaction, the mixture was poured into water (200 mL) and extracted with ethyl acetate (3× 50 mL). The ethyl acetate solution was washed with brine solution and water and dried over anhydrous sodium sulphate, filtered, and finally ethyl acetate was evaporated with a rotary evaporator. The crude product was purified by silica gel column chromatography using petroleum ether as the eluent to give title compound (4.7 g, 95 % yield) as a white solid. ^{1}H NMR (400MHz, $CDCl_3$) δ = 7.84 (s, 1 H), 7.60 - 7.47 (m, 2 H), 7.47 - 7.38 (m, 1 H), 7.11 (dd, *J* = 2.4, 8.8 Hz, 1 H), 7.00 (d, *J* = 2.3 Hz, 1 H), 3.98 (t, *J* = 6.4 Hz, 2 H), 1.88 - 1.70 (m, 2 H), 1.49 (qd, *J* = 7.3, 15.0 Hz, 2 H), 1.04 - 0.92 (m, 3 H). ^{13}C NMR (100MHz, $CDCl_3$) δ = 157.3, 133.0, 129.8, 129.5, 129.4, 128.3, 128.3, 120.0, 116.8, 106.4, 67.7, 31.2, 19.3, 13.8.

Synthesis of 2-(6-butoxynaphthalen-2-yl)-4,4,5,5-tetramethyl-1,3,2-dioxaborolane (7). 2-Bromo-6-butoxynaphthalene (2 g, 7 mmol), bis(pinacolato)diboron (1.8 g, 7.8 mmol), $PdCl_2$(dppf) (0.523 g, 0.7 mmol) and potassium acetate (2.1 g, 21 mmol) were added into a Schlenk tube under argon flow and then evacuated for 15 min. After this 1,4-dioxane (20 mL) was added to the above mixture under argon flow and the reaction mixture was stirred at room temperature for 30 minutes. When the mixture colour turned black, the reaction was heated to 80 ^{0}C for 24 h. The reaction was quenched by addition of water and extracted with ethyl acetate (3×50 mL). The ethyl acetate solution was dried over anhydrous sodium sulphate and filtered. Removal of ethyl acetate on rotary evaporator afforded the crude product, which was chromatographed on silica gel using petroleum ether: ethyl acetate (8:2, v/v) to give title compound (1.68 g, 72 % yield) as a white solid. ^{1}H NMR (400MHz, $CDCl_3$) δ = 8.29 (s, 1 H), 7.87 - 7.63 (m, 3 H), 7.17 - 7.04 (m, 2 H), 4.02 (t, *J* = 6.4 Hz, 2 H), 1.79 (dd, *J* = 6.0, 8.4 Hz, 2 H), 1.60 - 1.43 (m, 2 H), 1.36 (s, 12 H). ^{13}C NMR (100MHz, $CDCl_3$) δ = 158.0, 136.4, 135.9, 134.7, 131.0, 130.0, 128.2, 125.8, 118.9, 106.2, 83.6, 67.5, 31.2, 24.8, 19.2, 13.8.

Synthesis of 10-(4-methoxyphenyl)-10H-phenothiazine (1). The mixture of phenothiazine (5 g, 25 mmol), 4-iodoanisole (7.04 g, 30 mmol), copper powder (1.6g, 25 mmol), potassium carbonate (10.38 g, 75 mmol) and triethylene glycol dimethyl ether (TEGDME) (50 mL) was stirred into a Schlenk flask under nitrogen atmosphere at 180 ^{o}C for 24 h. The reaction mixture was cooled, filtered, and finally poured into ice cold water (500 mL). The brown precipitate was collected by filtration and dried. The product was recrystallized from ethyl acetate to give the title compound (5.74 g, 75 % yield) as a yellowish crystalline solid. ^{1}H NMR (400MHz, $CDCl_3$) δ = 7.35 - 7.29 (m, 2 H), 7.15 - 7.09 (m, 2 H), 7.00 (dd, *J* = 1.6, 7.5 Hz, 2 H), 6.88 - 6.76 (m, 4 H), 6.20 (dd, *J* = 1.0, 8.1 Hz, 2 H), 3.90 (s, 3 H).^{13}C NMR (100MHz, $CDCl_3$) δ = 159.2, 144.6, 133.2, 132.2, 126.8, 126.6, 122.2, 119.6, 115.8, 115.6, 55.5.

Synthesis of 10-(4-methoxyphenyl)-10H-phenothiazine-3-carbaldehyde (2). Dry N,N-dimethylformamide (3.04 mL) was taken into a 100 mL two-necked round bottom flask and

phosphorous oxychloride (3.65 mL) was added drop wise at 0 °C. The reaction mixture was stirred at room temperature for 30 min, and then the solution of 1 (4g, 13 mmol) in 1,2-dichloroethane (30 mL) was added and the mixture was heated to 80 ^oC for 12 h. The reaction mixture was poured into the water (100 mL), extracted with ethyl acetate (3x50 mL), and washed with brine solution and water. The ethyl acetate solution was dried over anhydrous sodium sulphate and filtered. Ethyl acetate was evaporated on rotary evaporator. The crude product was purified by silica gel column chromatography using petroleum ether: dichloromethane (7:3 v/v) as an eluent to give the title compound (3.7 g, 85 % yield) as a yellow solid. ^{1}H NMR (400MHz, $CDCl_3$) δ = 9.68 (s, 1 H), 7.44 (d, *J* = 2.1 Hz, 1 H), 7.30 - 7.24 (m, 3 H), 7.16 - 7.10 (m, 2 H), 6.95 (dd, *J* = 3.4, 6.1 Hz, 1 H), 6.86 - 6.80 (m, 2 H), 6.21 - 6.12 (m, 2 H), 3.90 (s, 3 H). ^{13}C NMR (100MHz, $CDCl_3$) δ = 189.7, 159.6, 149.5, 142.8, 132.2, 131.7, 130.8, 129.9, 127.4, 127.1, 126.6, 123.5, 120.0, 118.9, 116.4, 116.2, 114.9, 55.5.

Synthesis of 2-((10-(4-methoxyphenyl)-10H-phenothiazin-3-yl) methylene) malononitrile (O-1). 10-(4-Methoxyphenyl)-10H-phenothiazine-3-carbaldehyde (0.5g, 1.5 mmol) and malonitrile (0.148g, 2.2 mmol) were added into a two necked round bottom flask containing chloroform (10 mL), and refluxed for 12 h using catalytic amount of piperidine. The reaction mixture was poured into water, extracted using dichloromethane (3x50 mL), and washed with brine solution and water. The organic layer was dried over anhydrous sodium sulphate, filtered, and solvent was removed on rotary evaporator. The crude product was purified by column chromatography using petroleum ether: ethyl acetate (9:1) to gives the title compound (0.483 g, 87 % yield) as a red solid. ^{1}H NMR (400MHz, $CDCl_3$) δ = 7.43 (d, *J* = 2.1 Hz, 1 H), 7.40 - 7.34 (m, 2 H), 7.27 - 7.22 (m, 2 H), 7.17 - 7.10 (m, 2 H), 6.97 - 6.91 (m, 1 H), 6.88 - 6.81 (m, 2 H), 6.17 - 6.11 (m, 2 H), 3.91 (s, 3 H). ^{13}C NMR (100MHz, $CDCl_3$) δ = 159.9, 156.9, 149.6, 142.0, 131.6, 131.4, 131.3, 128.5, 127.3, 126.7, 125.2, 124.2, 120.5, 118.6, 116.8, 116.4, 115.3, 114.8, 113.6, 55.7.

Synthesis of 7-bromo-10-(4-methoxyphenyl)-10H-phenothiazine-3-carbaldehyde (3). 10-(4-Methoxyphenyl)-10H-phenothiazine-3-carbaldehyde (3 g, 8 mmol) was dissolved into a two necked round bottom flask in chloroform (50 mL). After cooling the mixture to 0° C for 30 min, previously prepared mixture of N-bromosuccinimide (1.92 g, 10.7 mmol) in chloroform (15 mL) was added drop wise into the flask. Then the reaction mixture was stirred overnight at room temperature. After completion of reaction, the reaction mixture was poured into water, extracted with chloroform (3×50 mL), and washed with brine solution and water. The chloroform solution was dried over sodium sulphate, filtered and chloroform was evaporated on a rotary evaporator. The crude product was purified on silica gel chromatography using petroleum ether: ethyl acetate (9:1, v/v) as an eluent to afford title compound (3.75 g, 85% yield) as a pale saffron solid. ^{1}H NMR (400MHz, $CDCl_3$) δ = 9.69 (s, 1 H), 7.43 (d, *J* = 1.7 Hz, 1 H), 7.28 (dd, *J* = 1.8, 8.7 Hz, 1 H), 7.27 - 7.22 (m, 2 H), 7.16 - 7.11 (m, 2 H), 7.06 (d, *J* = 2.2 Hz, 1 H), 6.90 (dd, *J* = 2.4, 8.8 Hz, 1 H), 6.19 (d, *J* = 8.6 Hz, 1 H), 5.99 (d, *J* = 8.8 Hz, 1 H), 3.91 (s, 3 H). ^{13}C NMR (100MHz, $CDCl_3$) δ = 189.6, 159.7, 149.0, 142.1, 131.9, 131.5, 131.1, 130.1, 129.7, 128.7, 127.4, 121.1, 119.2, 117.5, 116.4, 115.7, 115.1, 55.6.

Synthesis of 7,10-bis(4-methoxyphenyl)-10H-phenothiazine-3-carbaldehyde (4). Tetrahydrofuran (THF) (15 mL) was degassed into a Schlenk tube, using argon and 7-bromo-10-(4-methoxyphenyl)-10H-phenothiazine-3-carbaldehyde (1 g, 2.4 mmol), 4-methoxyphenylboronic acid (0.405 g, 2.6 mmol), 2 M aqueous solution of K_2CO_3 and $Pd(PPh_3)_4$ (280 mg) were added under argon flow in tetrahydrofuran and the reaction mixture was refluxed for 24 h. After completion of the reaction, the mixture was poured into water, extracted with ethyl acetate (3× 50 mL), and washed with brine solution and water. The combined organic layer was dried over anhydrous sodium sulphate, filtered and evaporated on a rotary evaporator. The crude product was purified using silica gel column chromatography with petroleum ether: ethyl acetate (19:1, v/v) as an eluent to give title compound (0.66 g, 66 % yield) as a saffron solid. ^{1}H NMR (400 MHz, $CDCl_3$) δ = 9.68 (s, 1 H), 7.46 (d, *J* = 2.0 Hz, 1 H), 7.39 (d, *J* = 8.8 Hz, 2 H), 7.31 - 7.26 (m, 3 H), 7.18 - 7.11 (m, 3 H), 6.99 (dd, *J* = 2.1, 8.7 Hz, 1 H), 6.92 (d, *J* = 8.8 Hz, 2 H), 6.18 (dd, *J* = 5.6, 8.6 Hz, 2 H), 3.91 (s, 3 H), 3.82 (s, 3 H). ^{13}C NMR (100 MHz, $CDCl_3$) δ = 189.7, 159.6, 159.1, 149.3, 141.4, 136.3, 132.3, 131.9, 131.6, 130.8, 129.9, 127.4, 127.3, 125.1, 124.5, 119.7, 119.3, 116.6, 116.2, 114.9, 114.2, 55.6, 55.3.

Synthesis of 2-((7,10-bis(4-methoxyphenyl)-10H-phenothiazin-3-) methylene) malononitrile (O-2). 7,10-Bis(4-methoxyphenyl)-10H-phenothiazine-3-carbaldehyde (0.5 g, 1.1 mmol) and malononitrile (112 mg, 1.7 mmol) were added into a two necked round bottom flask in $CHCl_3$ (10 mL), and refluxed for 14h using piperidine as catalyst. After completion of reaction, the reaction mixture was poured into water and extracted using dichloromethane (3 x 50 mL) and washed with brine solution and water. The organic layer was dried over anhydrous sodium sulphate, filtered and dichloromethane was removed on rotary evaporator. The crude was purified by column chromatography using petroleum ether: ethyl acetate (9:1, v/v) to obtain title compound (0.470 g, 85 % yield) as a red solid. ^{1}H NMR (400MHz, $CDCl_3$) δ = 7.43 (br. s., 1 H), 7.41 - 7.32 (m, 4 H), 7.26 (d, *J* = 8.2 Hz, 2 H), 7.19 - 7.09 (m, 3 H), 7.00 (d, *J* = 7.3 Hz, 1 H), 6.93 (d, *J* = 8.2 Hz, 2 H), 6.15 (dd, *J* = 8.7, 16.6 Hz, 2 H), 3.91 (s, 3 H), 3.83 (s, 3 H). ^{13}C NMR (100MHz, $CDCl_3$) δ = 159.9, 159.2, 156.8, 149.3, 140.5, 136.9, 131.6, 131.5, 131.3, 131.2, 128.5, 127.4, 125.2, 125.1, 124.5, 120.1, 119.0, 117.1, 116.4, 115.2, 114.8, 114.2, 113.6, 55.6, 55.3

Synthesis of 7-(6-butoxynaphthalen-2-yl)-10-(4-methoxyphenyl)-10H-phenothiazine-3-carbaldehyde (5). Tetrahydrofuran (15 mL) was degassed into a Schlenk tube, using argon and compound 7-bromo-10-(4-methoxyphenyl)-10H-phenothiazine-3-carbaldehyde (1 g, 2.4 mmol), 2-(6-butoxynaphthalen-2-yl)-4,4,5,5-tetramethyl-1,3,2-dioxaborolane (0.950 g, 3 mmol), 2 M aqueous solution of K_2CO_3 and $Pd(PPh_3)_4$ (0.280 g) were added under argon flow into tetrahydrofuran and the reaction mixture was heated to 60°C for 24 h. After completion of the reaction the reaction mixture was poured into water and was extracted with ethyl acetate (3× 50 mL), and washed with brine solution and water. The organic layer was dried over anhydrous sodium sulphate, filtered and evaporated on the rotary evaporator. The crude was purified using silica gel column chromatography with petroleum ether: ethyl acetate (5 %) as an eluent to give title compound (0.837 g, 65 % yield) as a saffron solid. ^{1}H NMR (400MHz, $CDCl_3$) δ = 9.69 (s, 1 H), 7.82 (d, *J* = 1.1 Hz, 1 H), 7.73 (d, *J* = 8.5 Hz, 2 H), 7.60 - 7.51 (m, 1 H), 7.47 (d, *J* = 1.9 Hz, 1 H), 7.36 - 7.24 (m, 4 H), 7.21 - 7.08 (m, 5 H), 6.21 (dd, *J* = 4.2, 8.5 Hz, 2 H), 4.08 (t, *J* =

6.5 Hz, 2 H), 3.92 (s, 3 H), 1.83 (dd, *J* = 6.1, 8.5 Hz, 2 H), 1.58 - 1.44 (m, 2 H), 1.06 - 0.95 (t, 3 H). ^{13}C NMR (100MHz, $CDCl_3$) δ = 189.7, 159.6, 157.2, 149.2, 141.7, 136.6, 134.3, 133.7, 132.2, 131.6, 130.8, 129.9, 129.5, 128.9, 127.4, 127.2, 125.5, 125.1, 124.9, 124.6, 119.7, 119.5, 119.4, 116.7, 116.3, 114.9, 106.3, 67.7, 55.5, 31.2, 19.3, 13.9.

Synthesis of 2-((7-(6-butoxynaphthalen-2-yl)-10-(4-methoxyphenyl)-10H-phenothiazin-3-yl) methylene) malononitrile (O-3). 7-(6-Butoxynaphthalen-2-yl)-10-(4-methoxyphenyl)-10H-phenothiazine-3-carbaldehyde (0.5 g, 0.94 mmol) and malonitrile (93 mg, 1.4 mmol) were added into a two necked round bottom flask containing $CHCl_3$ (10 mL), and refluxed for 12 h using catalytic amounts of piperidine. After completion of the reaction, the reaction mixture was extracted using dichloromethane (3 x 50 mL), washed with brine solution and water. The organic layer was dried over anhydrous sodium sulphate, filtered, and dichloromethane was removed on rotary evaporator. The crude was purified by column chromatography using petroleum ether: ethyl acetate (9:1, v/v) to give O-3 (0.455 g, 81 % yield) as a pale violet solid. ^{1}H NMR (400MHz, $CDCl_3$) δ = 7.82 (s, 1 H), 7.74 (dd, *J* = 2.1, 8.5 Hz, 2 H), 7.55 (dd, *J* = 1.5, 8.5 Hz, 1 H), 7.44 (d, *J* = 2.1 Hz, 1 H), 7.41 - 7.35 (m, 2 H), 7.30 - 7.24 (m, 3 H), 7.19 - 7.13 (m, 4 H), 7.12 (d, *J* = 2.1 Hz, 1 H), 6.22 (d, *J* = 8.5 Hz, 1 H), 6.15 (d, *J* = 8.9 Hz, 1 H), 4.08 (t, *J* = 6.6 Hz, 2 H), 3.92 (s, 3 H), 1.88 - 1.79 (m, 2 H), 1.56 - 1.49 (m, 2 H), 1.01 (t, *J* = 7.3 Hz, 3 H). ^{13}C NMR (100MHz, $CDCl_3$) δ = 160.0, 157.4, 156.8, 149.3, 140.8, 137.3, 134.1, 133.9, 131.6, 131.3, 129.5, 129.6, 129.0, 128.6, 127.3, 125.8, 125.2, 125.0, 125.0, 124.8, 120.2, 119.7, 119.1, 117.2, 116.5, 115.3, 114.8, 113.6, 106.3, 67.8, 55.7, 31.3, 19.3, 13.9.

Computational Details

All calculations were carried out using the Gaussian09[1] software package without symmetry constrains. Solvent effects (chloroform) were considered in every calculation using the Polarizable Continuum Model (PCM) initially devised by Tomasi and coworkers[2-4] as implemented on Gaussian 09, with radii and non-electrostatic terms for Truhlar and coworkers' SMD solvation model.[5] Density Functional Theory (DFT) and Time-Dependent DFT (TD-DFT) were used for computation of the ground and excited state properties of the phenothiazines, respectively. All calculations have been performed using the PBE1PBE functional and 6-31G(d, p)[6-10] basis set. That functional uses a hybrid generalised gradient approximation (GGA), including 25% mixture of Hartree-Fock[11] exchange with DFT[12] exchange-correlation, given by Perdew, Burke and Ernzerhof functional (PBE).[13,14]

Extensive TD-DFT calculations studies with different functionals have demonstrated that global hybrid methods containing between 22-25% of exact exchange provide better match with reference data.[15] Vertical excitation calculations of compound **O-1** with PBE1PBE, CAM-B3LYP and B3LYP functionals and 6-31G(d, p) basis set using PCM were compared with the experimental UV spectrum in chloroform. PBE1PBE was adopted for the computational study since the λ_{max} calculated (494 nm) was in considerable better agreement with the experimental value (490 nm) than the one obtained with CAM-B3LYP (400 nm) or B3LYP (516 nm). Successful uses of PBE1PBE in the studies of vertical excitation systems have been reported for aromatic[16-18] and push-pull systems.[19,20]

The computational data described was obtained by using the following computational protocol: 1) The ground state geometry of each molecule has been fully optimized with default cut-offs on forces and step size to determine convergence. 2) The analytical calculation of the vibrational frequencies at the same level of theory verified the optimized structure by checking that they corresponded to true minima of the potential energy surface by the absence of imaginary frequencies. 3) The first six low-lying excited states have been determined within the vertical TD-DFT, with the default non-equilibrium solvation procedure. The less energetic allowed transition ($S_0 \rightarrow S_1{}^*$) were mainly composed by HOMO→LUMO transitions. 4) The minimum energy point on the excited state potential energy surface was calculated by a TD-DFT geometry optimization of the first excited state in solution, with tight convergence criteria, with equilibrium, linear response solvation. 5) A Natural Population Analysis (NPA)[21-28] was performed as implemented on Gaussian 09 to study the electronic structure of the optimised species.

Computational analysis

The electronic nature of the newly synthesised phenothiazines **O-1** and **O-2** was explored by Density Functional Theory methods, as well as **O-3** simplified by replacement of the *n*-butyl chain with a methyl (**O-3(Me)**). In the ground state the geometries of the phenothiazines are slightly bent in the phenothiazine heterocyclic ring and adopt a butterfly shape. The presence of different substituents at the C(7) position of the phenothiazine has little or no effect on the dihedral angles made by the two aromatic rings, being about 19-20° for the three molecules studied. Although alkyl *N*-substituted phenothiazines present larger dihedral angles (*ca*. 41°), the presence of the methoxyphenyl *N*-substituent induces the heterocyclic moiety to adopt a geometry closer to planarity.[29] On the other hand, the methoxyphenyl *N*-substituent preferentially adopts a perpendicular position to the unsubstituted phenyl ring in **O-1** (Φ_1) and its co-planarity demands for 7 kcal/mol (Fig. S16). Regarding rotation of substituents at C(7) position, these adopt a non-planar geometry having a dihedral angle (Φ_2) of ca. 34° with the phenothiazine ring system in **O-2** and **O-3(Me)** (Fig. S16). Such slight distortion does not seem to encumber the resonance between the substituents and the phenothiazine.

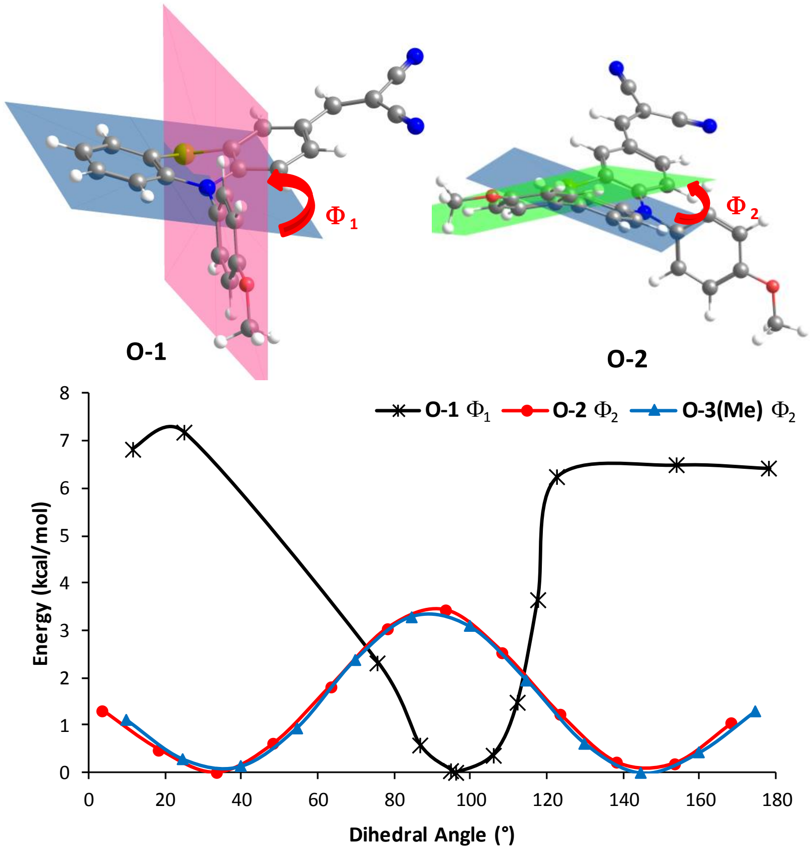

Figure S16. Plane figures of phenothiazines optimized structures, dihedral angles and energy profiles for substituents rotation

The electron density distribution in the LUMO of the phenothiazines is mainly localized on the malononitrile end group and the adjacent phenyl ring, while in **O-2** and **O-3(Me)** HOMO orbitals are delocalized over the phenothiazine moiety and its C(7) substituents, as shown in Fig. 7. Clearly, introduction of electron rich substituents in the C(7) position of the phenothiazine increases their HOMO energy while keeping the energy of LUMO orbitals localized on the acceptor end. The methoxyphenyl *N*-substituent is not involved in the electron density of the frontier orbitals, as would be expected due to its perpendicularity with the phenothiazine system. The strongest transitions ($S_0 \rightarrow S_1$) are visibly dominated by HOMO→LUMO transitions for the three studied molecules, as determined by TDDFT (Table S1). In the case of phenothiazine **O-3(Me)** a small contribution from HOMO-1→LUMO transition was found. The λ_{max} found reflects the observed experimental trends, although the absolute values for the excitation energies are overestimated by the method (by *ca.* 0.9-1.1 eV). Further inspection into the S_1 state and the distribution of natural charges in S_0 compared to S_1 (Fig. S17) confirms that the three systems behave similarly concerning the acceptor moiety and the methoxyphenyl *N*-substituent. The acceptor moiety is electronically enriched by 0.35 upon excitation, while the *N*-substituent barely changes its natural charge. On the other hand, while most of the charge transfer occurs from the phenothiazine ring into the malononitrile, the electron rich substituent at C(7) contributes to some extent to the internal charge transfer. In such instance the 6-methoxynaphtalene of **O-3(Me)** is slightly more

electron donating than the 4-methoxyphenyl substituent in **O-2**, as demonstrated by the changes in natural charges (0.09 for **O-2** and 0.12 for **O-3(Me)**).

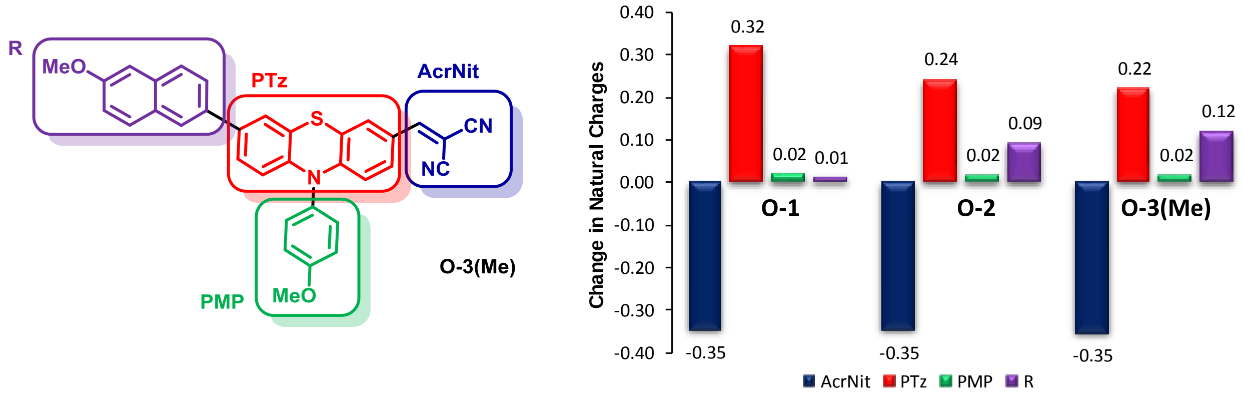

Figure S17. Differences in Natural Charges between ground state (S_0) and excited state (S_1), estimated by a time dependent DFT/PBE1PBE model.

Table S1. Selected parameters for the vertical excitation of compounds **O-1**, **O-2** and **O-3(Me)** (only transitions with f > 0.1 are presented). Electronic excitation energies (eV), oscillator strengths (f) and configurations of the 6 low-lying excited states. Calculated by TDDFT//PBE1PBE/6-31G(d,p), based on the optimized ground state geometries (chloroform was employed as solvent in every calculations).

		**TDDFT//PBE1PBE/6-31G(d,p)**			
**Compound**	**Electronic Transition**[a]	**Excitation Energy**	f^b	**Composition**[c]	**CI**[d]
**O-1**	$S_0 \rightarrow S_1$	2.51 eV (494 nm)	0.4296	H → L	0.69897 (98 %)
	$S_0 \rightarrow S_3$	3.57 eV (348 nm)	0.4483	H−2 → L	0.67071 (90 %)
				H → L+1	0.16637 (6 %)
	$S_0 \rightarrow S_4$	3.91 eV (317 nm)	0.3605	H → L+1	0.65235 (85 %)
				H−2 → L	0.16726 (6 %)
				H → L+3	0.13919 (4 %)
	$S_0 \rightarrow S_5$	4.24 eV (293 nm)	0.1968	H−3 → L	0.64701 (84 %)
				H → L+2	0.22166 (10 %)
				H → L+4	0.10537 (2 %)
**O-2**	$S_0 \rightarrow S_1$	2.39 eV (518 nm)	0.5523	H → L	0.69641 (97 %)
	$S_0 \rightarrow S_4$	3.62 eV (342 nm)	0.3657	H−3 → L	0.57008 (65 %)
				H → L+1	0.35145 (25 %)
				H−1 → L	0.16840 (6 %)
	$S_0 \rightarrow S_5$	3.82 eV (325 nm)	0.4415	H → L+1	0.54958 (60 %)
				H−3 → L	0.35183 (25 %)
				H → L+2	0.15250 (5 %)
				H−1 → L	0.10189 (2 %)
				H−1 → L+1	0.10135 (2 %)
	$S_0 \rightarrow S_6$	4.06 eV (305 nm)	0.2992	H → L+3	0.61455 (76 %)
				H → L+4	0.22405 (10 %)
				H → L+2	0.14733 (4 %)
**O-3(Me)**	$S_0 \rightarrow S_1$	2.34 eV (520 nm)	0.5904	H → L	0.68775 (94 %)
				H−1 → L	0.13211 (3 %)
	$S_0 \rightarrow S_3$	3.52 eV (352 nm)	0.3264	H−2 → L	0.47201 (45 %)
				H → L+1	0.30403 (18 %)
				H−4 → L	0.28544 (16 %)
				H−3 → L	0.25604 (13 %)
				H → L+2	0.13067 (3 %)
	$S_0 \rightarrow S_4$	3.55 eV (350 nm)	0.1908	H−2 → L	0.51881 (54 %)
				H−3 → L	0.29695 (18 %)
				H → L+1	0.28020 (16 %)
				H−4 → L	0.20877 (9 %)
				H → L+2	0.10212 (2 %)
	$S_0 \rightarrow S_5$	3.66 eV (339 nm)	0.1475	H → L+1	0.53458 (57 %)
				H−3 → L	0.33839 (23 %)
				H−4 → L	0.26222 (14 %)
				H−1 → L	0.10157 (2 %)

[a] Only excited states with f>0.100 were considered. [b] Oscillator strength [c] H stands for HOMO and L stands for LUMO [d] Absolute CI coefficient of the wavefunction for each excitation. In parenthesis are indicated the percentage contribution of the configuration to excitation.

Atomic coordinates for all the optimized species
DFT-PBE1PBE/6-31G** for ground state geometries

O-1

6	2.936421	-1.015454	0.805806
6	1.926595	-1.983083	0.973942
6	0.718709	-1.872942	0.317370
6	0.448309	-0.801959	-0.558518
6	1.480844	0.139102	-0.776542
6	2.673845	0.045488	-0.087063
7	-0.782368	-0.682440	-1.186531
6	-1.280289	0.560483	-1.650461
6	-0.413660	1.606778	-2.004461
16	1.332805	1.342232	-2.062057
6	-2.658101	0.780703	-1.759994
6	-3.153179	2.003008	-2.204395
6	-2.288115	3.041961	-2.525977
6	-0.916088	2.839439	-2.413420
1	2.072298	-2.830575	1.632804
1	-0.038102	-2.629458	0.483051
1	3.442096	0.795217	-0.258847
1	-3.350371	-0.011579	-1.502221
1	-4.227602	2.136855	-2.287214
1	-0.221050	3.635900	-2.663948
6	4.215328	-1.016469	1.453530
6	4.764093	-1.877957	2.369466
1	4.865161	-0.189478	1.175214
6	4.126982	-3.033366	2.904985
7	3.624876	-3.980879	3.355013
6	6.082799	-1.631015	2.853930
7	7.157654	-1.430840	3.249435
6	-1.803688	-2.691713	-2.117363
6	-1.710202	-1.773676	-1.078600
6	-2.518859	-1.921312	0.053144
6	-3.408663	-2.978308	0.136591
6	-3.505362	-3.904324	-0.912396
6	-2.696797	-3.758920	-2.043681
8	-4.403445	-4.895831	-0.735415
1	-2.751668	-4.464233	-2.865119
1	-1.172302	-2.571954	-2.992992
1	-2.445836	-1.203610	0.865259
1	-4.042632	-3.108477	1.008071
6	-4.542276	-5.860552	-1.763724
1	-5.309269	-6.556965	-1.421699
1	-4.867572	-5.401441	-2.704748
1	-3.607744	-6.409612	-1.929118
1	-2.669576	4.000259	-2.864106

O-2

6	2.825910	-0.749732	0.644215
6	1.926082	-1.808216	0.878333
6	0.685037	-1.833618	0.277437
6	0.268013	-0.814391	-0.603636
6	1.192592	0.216741	-0.891292
6	2.417762	0.257633	-0.256141
7	-0.995556	-0.832379	-1.171077
6	-1.623584	0.331453	-1.675364
6	-0.881309	1.440684	-2.107833
16	0.881348	1.363345	-2.198536
6	-3.017856	0.416617	-1.754045
6	-3.642622	1.554191	-2.248829
6	-2.908845	2.672434	-2.658924
6	-1.513720	2.592218	-2.561071
1	2.186689	-2.621372	1.545081
1	0.015723	-2.657276	0.492182
1	3.099194	1.074160	-0.480554
1	-3.625561	-0.425065	-1.443664
1	-4.725641	1.557688	-2.327371
1	-0.901454	3.443815	-2.844210
6	4.125929	-0.606598	1.230237
6	4.800955	-1.382549	2.138791
1	4.676391	0.272623	0.901405
6	4.310249	-2.579570	2.733797
7	3.928889	-3.558818	3.232032
6	6.108596	-0.989642	2.551585
7	7.175049	-0.670569	2.887417
6	-1.885387	-2.941057	-2.011117
6	-1.811041	-2.000976	-0.990607
6	-2.533828	-2.198342	0.190114
6	-3.314947	-3.331069	0.343168
6	-3.391448	-4.280443	-0.686334
6	-2.673543	-4.081286	-1.869476
8	-4.180684	-5.347007	-0.440636
1	-2.717297	-4.801678	-2.678303
1	-1.322409	-2.780626	-2.926019
1	-2.478006	-1.461263	0.986093
1	-3.879902	-3.502141	1.254184
6	-4.306752	-6.330329	-1.452823
1	-4.981854	-7.088363	-1.052820
1	-4.740015	-5.911745	-2.368928
1	-3.341617	-6.795858	-1.685074
6	-2.968652	4.662316	-4.182195
6	-3.568337	3.885097	-3.185288
6	-4.823777	4.298813	-2.707945
6	-5.445570	5.431187	-3.205427
6	-4.829069	6.195889	-4.204086
6	-3.579705	5.804593	-4.692079
8	-5.514798	7.282517	-4.626597
1	-3.081843	6.370653	-5.471358
1	-2.009807	4.360550	-4.594879
1	-5.312329	3.736310	-1.917115
1	-6.411676	5.750676	-2.825995
6	-4.926917	8.089300	-5.630331
1	-5.631740	8.901586	-5.815351
1	-3.971000	8.512675	-5.299046
1	-4.773311	7.529215	-6.560697

O-3(Me)

6	2.998071	-5.662209	6.368868

6	2.081092	-6.695072	6.646563
6	0.856216	-6.748635	6.014995
6	0.475688	-5.786555	5.057287
6	1.416055	-4.781810	4.731656
6	2.624298	-4.710067	5.396368
7	-0.770628	-5.832312	4.452702
6	-1.377411	-4.693952	3.872061
6	-0.615751	-3.616189	3.395952
16	1.147685	-3.718019	3.346778
6	-2.769111	-4.603890	3.757324
6	-3.372746	-3.490183	3.189039
6	-2.620182	-2.400794	2.737553
6	-1.227929	-2.486716	2.865438
1	2.313793	-7.463379	7.373987
1	0.172358	-7.550496	6.263805
1	3.319286	-3.914776	5.139626
1	-3.390206	-5.423749	4.098039
1	-4.453402	-3.481765	3.083934
1	-0.601716	-1.658400	2.546344
6	4.285942	-5.496226	6.976158
6	4.940922	-6.234927	7.929165
1	4.845710	-4.632937	6.621842
6	4.440222	-7.410791	8.557516
7	4.050734	-8.372986	9.081929
6	6.239833	-5.825143	8.353346
7	7.299057	-5.492581	8.698712
6	-1.661551	-7.967338	3.686135
6	-1.598881	-6.985965	4.667847
6	-2.354587	-7.122678	5.836553
6	-3.156537	-8.236745	6.016729
6	-3.222507	-9.226881	5.025633
6	-2.470346	-9.089144	3.855004
8	-4.037414	-10.268823	5.292609
1	-2.503678	-9.842719	3.076367
1	-1.074701	-7.852411	2.779443
1	-2.309638	-6.352431	6.601330
1	-3.747547	-8.360764	6.918795
6	-4.161476	-11.284636	4.312794
1	-4.869225	-12.009065	4.718648
1	-4.555176	-10.888147	3.369484
1	-3.203471	-11.784892	4.127941
6	-2.614214	-0.490404	1.095048
6	-3.260447	-1.209746	2.141444
6	-4.495579	-0.766738	2.575311
6	-5.120734	0.366418	2.002867
6	-4.463835	1.077658	0.952030
6	-3.193217	0.610807	0.521170
6	-5.088476	2.202913	0.377244
1	-2.686621	1.136155	-0.284476
1	-1.653623	-0.839515	0.726562
1	-5.003898	-1.277930	3.389435
6	-6.384541	0.831278	2.438960
6	-6.327064	2.626729	0.821004
6	-6.981100	1.930587	1.868575
1	-6.891482	0.301144	3.241406
1	-4.599132	2.748809	-0.424531
8	-6.848697	3.714526	0.206200
6	-8.128544	4.168205	0.606692
1	-7.951201	2.258176	2.225148
1	-8.349617	5.031732	-0.023101
1	-8.138801	4.481772	1.657294
1	-8.899312	3.404617	0.446419

References

1) M. J. Frisch, G. W. Trucks, H. B. Schlegel, G. E. Scuseria, M. A. Robb, J. R. Cheeseman, G. Scalmani, V. Barone, B. Mennucci, G. A. Petersson, H. Nakatsuji, M. Caricato, X. Li, H. P. Hratchian, A. F. Izmaylov, J. Bloino, G. Zheng, J. L. Sonnenberg, M. Hada, M. Ehara, K. Toyota, R. Fukuda, J. Hasegawa, M. Ishida, T. Nakajima, Y. Honda, O. Kitao, H. Nakai, T. Vreven, J. A. Montgomery, Jr., J. E. Peralta, F. Ogliaro, M. Bearpark, J. J. Heyd, E. Brothers, K. N. Kudin, V. N. Staroverov, T. Keith, R. Kobayashi, J. Normand, K. Raghavachari, A. Rendell, J. C. Burant, S. S. Iyengar, J. Tomasi, M. Cossi, N. Rega, J. M. Millam, M. Klene, J. E. Knox, J. B. Cross, V. Bakken, C. Adamo, J. Jaramillo, R. Gomperts, R. E. Stratmann, O. Yazyev, A. J. Austin, R. Cammi, C. Pomelli, J. W. Ochterski, R. L. Martin, K. Morokuma, V. G. Zakrzewski, G. A. Voth, P. Salvador, J. J. Dannenberg, S. Dapprich, A. D. Daniels, O. Farkas, J. B. Foresman, J. V. Ortiz, J. Cioslowski, and D. J. Fox, Gaussian 09, revision D.01; Gaussian Inc.: Wallingford CT, 2013.
2) E. Cancès, B. Mennucci, J. Tomasi, A new integral equation formalism for the polarizable continuum model: Theoretical background and applications to isotropic and anisotropic dielectrics. *J. Chem. Phys.* 1997, **107**(8)**:** 3032-3041.
3) B. Mennucci, J. Tomasi, Continuum solvation models: A new approach to the problem of solute's charge distribution and cavity boundaries. *J. Chem. Phys.* 1997, **106**(12)**:** 5151-5158.
4) M. Cossi, V. Barone, B. Mennucci, J. Tomasi, Ab initio study of ionic solutions by a polarizable continuum dielectric model. *Chem. Phys. Lett.* 1998, **286**(3–4)**:** 253-260.
5) A. V. Marenich, C. J. Cramer, D. J. Truhlar, Universal Solvation Model Based on Solute Electron Density and on a Continuum Model of the Solvent Defined by the Bulk Dielectric Constant and Atomic Surface Tensions. *J. Phys. Chem. B* 2009, **113**(18)**:** 6378-6396.
6) R. Ditchfield, W. J. Hehre, J. A. Pople, Self-Consistent Molecular-Orbital Methods. IX. An Extended Gaussian-Type Basis for Molecular-Orbital Studies of Organic Molecules. *J. Chem. Phys.* 1971, **54**(2)**:** 724-728.
7) W. J. Hehre, R. Ditchfield, J. A. Pople, Self—Consistent Molecular Orbital Methods. XII. Further Extensions of Gaussian—Type Basis Sets for Use in Molecular Orbital Studies of Organic Molecules. *J. Chem. Phys.* 1972, **56**(5)**:** 2257-2261.
8) P. C. Hariharan, J. A. Pople, Accuracy of AH n equilibrium geometries by single determinant molecular orbital theory. *Mol. Phys.* 1974, **27**(1)**:** 209-214.
9) M. S. Gordon, The isomers of silacyclopropane. *Chem. Phys. Lett.* 1980, **76**(1)**:** 163-168.
10) P. C. Hariharan, J. A. Pople, The influence of polarization functions on molecular orbital hydrogenation energies. *Theoret. Chim. Acta* 1973, **28**(3)**:** 213-222.
11) W. J. Hehre, L. Radom, P. v. R. Schleye, J. Pople *Ab initio molecular orbital theory*. John Wiley & Sons: New York, 1986.
12) R. G. Parr, W. Yang *Density Functional Theory of Atoms and Molecules*. Oxford University Press: New York, 1989.
13) J. P. Perdew Density-functional approximation for the correlation energy of the inhomogeneous electron gas. *Phys. Rev. B.* 1986, **33**(12)**:** 8822-8824.
14) J. P. Perdew, K. Burke, M. Ernzerhof, Generalized Gradient Approximation Made Simple [Phys. Rev. Lett. 77, 3865 (1996)]. *Phys. Rev. Lett.* 1997, **78**(7)**:** 1396-1396.
15) D. Jacquemin, V. Wathelet, E. A. Perpète, C. Adamo, Extensive TD-DFT Benchmark: Singlet-Excited States of Organic Molecules. *J. Chem. Theory Comput.* 2009, **5**(9)**:** 2420-2435.
16) D. Jacquemin, E. A. Perpète, G. E. Scuseria, I. Ciofini, C. Adamo, TD-DFT Performance for the Visible Absorption Spectra of Organic Dyes: Conventional versus Long-Range Hybrids. *J. Chem. Theory Comput.* 2008, **4**(1)**:** 123-135.

17) D. Bousquet, R. Fukuda, P. Maitarad, D. Jacquemin, I. Ciofini, C. Adamo, M. Ehara, Excited-State Geometries of Heteroaromatic Compounds: A Comparative TD-DFT and SAC-CI Study. *J. Chem. Theory Comput.* 2013, **9**(5)**:** 2368-2379.
18) Y. Xue, Y. Liu, L. An, L. Zhang, Y. Yuan, J. Mou, L. Liu, Y. Zheng, Electronic structures and spectra of quinoline chalcones: DFT and TDDFT-PCM investigation. *Comp. Theor. Chem.* 2011, **965**(1)**:** 146-153.
19) C. A. Guido, P. Cortona, B. Mennucci, C. Adamo, On the Metric of Charge Transfer Molecular Excitations: A Simple Chemical Descriptor. *J. Chem. Theory Comput.* 2013, **9**(7)**:** 3118-3126.
20) L. Zhang, K. Pei, M. Yu, Y. Huang, H. Zhao, M. Zeng, Y. Wang, J. Gao, Theoretical Investigations on Donor–Acceptor Conjugated Copolymers Based on Naphtho[1,2-c:5,6-c]bis[1,2,5]thiadiazole for Organic Solar Cell Applications. *J. Phys. Chem. C* 2012, **116**(50)**:** 26154-26161.
21) J. E. Carpenter, PhD thesis, University of Wisconsin Madison WI, 1987.
22) J. E. Carpenter, F. Weinhold, Analysis of the Geometry of the Hydroxymethyl Radical by the Different Hybrids for Different Spins Natural Bond Orbital Procedure. *Theochem-J. Mol. Struct.* 1988, **46:** 41-62.
23) J. P. Foster, F. Weinhold, Natural hybrid orbitals. *J. Am. Chem. Soc.* 1980, **102**(24)**:** 7211-7218.
24) A. E. Reed, F. Weinhold, Natural Bond Orbital Analysis of near-Hartree-Fock Water Dimer. *J. Chem. Phys.* 1983, **78**(6)**:** 4066-4073.
25) A. E. Reed, F. Weinhold, Natural Localized Molecular-Orbitals. *J. Chem. Phys.* 1985, **83**(4)**:** 1736-1740.
26) A. E. Reed, R. B. Weinstock, F. Weinhold, Natural-Population Analysis. *J. Chem. Phys.* 1985, **83**(2)**:** 735-746.
27) A. E. Reed, L. A. Curtiss, F. Weinhold, Intermolecular Interactions from a Natural Bond Orbital, Donor-Acceptor Viewpoint. *Chem. Rev.* 1988, **88**(6)**:** 899-926.
28) F. Weinhold, J. E. Carpenter, *The Structure of Small Molecules and Ions*. Plenum, 1988.
29) S. H. Kim, C. Sakong, J. B. Chang, B. Kim, M. J. Ko, D. H. Kim, K. S. Hong and J. P. Kim, *Dye. Pigment.*, 2013, **97**, 262–271.
